# A Paleogenomic Reconstruction of the Deep Population History of the Andes

**DOI:** 10.1016/j.cell.2020.04.015

**Published:** 2020-05-28

**Authors:** Nathan Nakatsuka, Iosif Lazaridis, Chiara Barbieri, Pontus Skoglund, Nadin Rohland, Swapan Mallick, Cosimo Posth, Kelly Harkins-Kinkaid, Matthew Ferry, Éadaoin Harney, Megan Michel, Kristin Stewardson, Jannine Novak-Forst, José M. Capriles, Marta Alfonso Durruty, Karina Aranda Álvarez, David Beresford-Jones, Richard Burger, Lauren Cadwallader, Ricardo Fujita, Johny Isla, George Lau, Carlos Lémuz Aguirre, Steven LeBlanc, Sergio Calla Maldonado, Frank Meddens, Pablo G. Messineo, Brendan J. Culleton, Thomas K. Harper, Jeffrey Quilter, Gustavo Politis, Kurt Rademaker, Markus Reindel, Mario Rivera, Lucy Salazar, José R. Sandoval, Calogero M. Santoro, Nahuel Scheifler, Vivien Standen, Maria Ines Barreto, Isabel Flores Espinoza, Elsa Tomasto-Cagigao, Guido Valverde, Douglas J. Kennett, Alan Cooper, Johannes Krause, Wolfgang Haak, Bastien Llamas, David Reich, Lars Fehren-Schmitz

**Affiliations:** 1 Department of Genetics, Harvard Medical School, Boston, Massachusetts 02115, USA; 2 Harvard-MIT Division of Health Sciences and Technology, Boston, MA 02115, USA; 3 Max Planck Institute for the Science of Human History, Jena 07745, Germany; 4 Department of Evolutionary Biology and Environmental Studies, University of Zurich, Zurich 8057, Switzerland; 5 Francis Crick Institute, London NW1 1AT, UK; 6 Howard Hughes Medical Institute, Harvard Medical School, Boston, MA 02446, USA; 7 Broad Institute of Harvard and MIT, Cambridge, MA 02142, USA; 8 UCSC Paleogenomics, University of California, Santa Cruz, Santa Cruz, CA 95064, USA; 9 Department of Anthropology, The Pennsylvania State University, University Park, PA 16802, USA; 10 Department of Sociology, Anthropology and Social Work, Kansas State University, Manhattan, KS 66506, USA; 11 Sociedad de Arqueología de La Paz, 5294 La Paz, Bolivia; 12 McDonald Institute for Archaeological Research, University of Cambridge, Downing St., Cambridge, CB2 3ER, UK; 13 Department of Anthropology, Yale University, New Haven, CT 06511, USA; 14 Office of Scholarly Communication, Cambridge University Library, Cambridge CB3 9DR, UK; 15 Centro de Genética y Biología Molecular, Facultdad de Medicina, Universidad de San Martín de Porres, Lima 15011, Peru; 16 Peruvian Ministry of Culture, DDC Ica, Directos of the Nasca-Palpa Management Plan, Calle Juan Matta 880, Nasca 11401, Peru; 17 Sainsbury Research Unit, University of East Anglia, Norwich Research Park, Norwich NR4 7TJ, UK; 18 Carrera de Arqueología, Universidad Mayor de San Andrés, Edificio Facultad de Ciencias Sociales 3er Piso, La Paz 1995, Bolivia; 19 Harvard Peabody Museum, Harvard University, Cambridge, MA 02138, USA; 20 School of Archaeology, Geography and Environmental Sciences, University of Reading, Reading, Berkshire, RG6 6AH, UK; 21 INCUAPA-CONICET, Facultad de Ciencias Sociales, Universidad Nacional del Centro de la Provincia de Buenos Aires, Olavarría 7400, Argentina; 22 Institutes for Energy and the Environment, The Pennsylvania State University, University Park, PA 16802, USA; 23 Department of Anthropology, The Pennsylvania State University, University Park, PA 16802, USA; 24 Department of Anthropology, Michigan State University, East Lansing, MI 48824, USA; 25 Commission for Archaeology of Non-European Cultures, German Archaeological Institute, Berlin 14195, Germany; 26 Universidad de Magallanes, Punta Arenas 6210427, Chile; 27 Field Museum Natural History 1400 S Lake Shore Dr., Chicago, IL 60605, USA; 28 Instituto de Alta Investigation, Universidad de Tarapaca, Antafogasta 1520, Arica, 1000000, Chile; 29 Departamento de Antropología, Universidad de Tarapacá, Antafogasta 1520, Arica, 1000000, Chile; 30 Museo de Sitio Huaca Pucllana, Calle General Borgoño, Cuadra 8, Miraflores, Lima 18, Peru; 31 Department of Humanities, Pontifical Catholic University of Peru, San Miguel 15088, Peru; 32 Australian Centre for Ancient DNA, School of Biological Sciences and The Environment Institute, Adelaide University, Adelaide, SA 5005, Australia; 33 Department of Anthropology, University of California, Santa Barbara, Santa Barbara, CA 93106, USA; 34 Department of Human Evolutionary Biology, Harvard University, Cambridge, MA 02138, USA; 35 UCSC Genomics Institute, University of California, Santa Cruz, Santa Cruz, CA 95064, USA

**Keywords:** Andes, population genetics, archaeology, anthropology, ancient DNA

## Abstract

There are many unanswered questions about the population history of the Central and South Central Andes, particularly regarding the impact of large-scale societies, such as the Moche, Wari, Tiwanaku, and Inca. We assembled genome-wide data on 89 individuals dating from ∼9,000-500 years ago (BP), with a particular focus on the period of the rise and fall of state societies. Today’s genetic structure began to develop by 5,800 BP, followed by bi-directional gene flow between the North and South Highlands, and between the Highlands and Coast. We detect minimal admixture among neighboring groups between ∼2,000–500 BP, although we do detect cosmopolitanism (people of diverse ancestries living side-by-side) in the heartlands of the Tiwanaku and Inca polities. We also highlight cases of long-range mobility connecting the Andes to Argentina and the Northwest Andes to the Amazon Basin.

**Video Abstract:**

## Highlights

• Ancient DNA transect reveals north-south substructure of Andean highlands by 5,800 BP
• After 5,800 BP, gene flow mixed highland people with their neighbors
• After 2,000 BP, striking genetic continuity through rise and fall of major cultures

## Graphical Abstract

**Figure:**
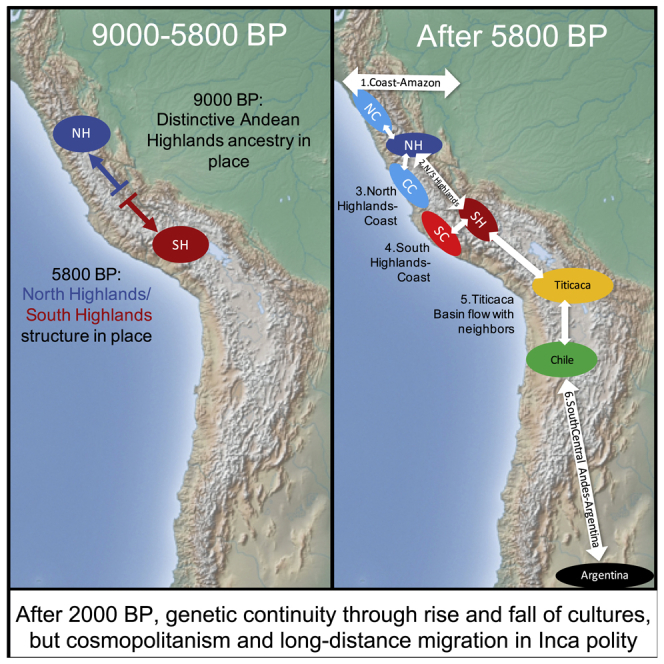

## Teaser

Genome-wide data from 89 ancient humans illuminates the changes to the genetic landscape in the Central Andes over 9,000 years, revealing large-scale gene flow and cosmopolitan societies in the Tiwanaku and Inca polities.

## Introduction

The South American Andean regions have a long and dynamic history beginning with the arrival of the first hunter-gatherers at least ∼14,500 BP. In the Central and South-Central Andean regions (present-day Peru, Bolivia, and North Chile), early settlements in both the Coast and the Highlands (Capriles et al., 2016, Capriles et al., 2016b, Chala-Aldana et al., 2018, Dillehay, 2017, Rademaker et al., 2014, Santoro et al., 2019) were followed by the development of sedentary lifestyles, complex societies, and eventually archaeological cultures with wide spheres of influence, such as the Wari (∼1,400–950 BP), Tiwanaku (∼1,400–950 BP), and Inca (∼510–420 BP) (BP: before present, defined as years before 1950 CE; in what follows, all radiocarbon dates are corrected with appropriate calibration curves as justified in STAR Methods and summarized by the midpoint of their estimated date ranges rounded to the closest century; Table S1).

Archaeological research in the Central Andes is extraordinarily rich (Silverman and Isbell, 2008), but ancient DNA (aDNA) studies to date have been limited, so there has been little information about demographic change over time. Studies of uniparental DNA indicated evidence for a degree of genetic homogeneity of the Central and Southern Highlands, especially for the Y chromosome (Barbieri et al., 2014, Gómez-Carballa et al., 2018, Harris et al., 2018, Sandoval et al., 2013, Sandoval et al., 2016), while studies with aDNA suggested substantial continuity as well as gene flow between the Coast and the Highlands (Baca et al., 2012, Fehren-Schmitz et al., 2014, Fehren-Schmitz et al., 2017, Llamas et al., 2016, Russo et al., 2018, Valverde et al., 2016). High coverage genome-wide ancient DNA data from South America from the time before European contact began to be published in 2018, with most data from mid- to early-Holocene hunter-gatherers (in the Central and South-Central Andes, 23 individuals were reported) (Lindo et al., 2018, Moreno-Mayar et al., 2018b, Posth et al., 2018). Although these studies had large geographic and temporal gaps, they were critical in showing that individuals from the Central and South-Central Andes up to at least ∼9,000 BP are more closely related to modern Andean highland, rather than coastal or Amazonian populations (Lindo et al., 2018, Posth et al., 2018). An additional lineage was found to have begun spreading in this region by at least ∼4,200 BP (Posth et al., 2018) and had a significant genetic affinity (excess allele sharing) with groups in Mexico and the California Channel Islands.

**Figure 1:**
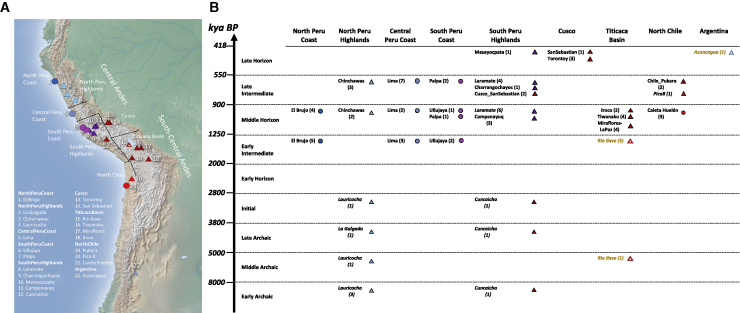
Distribution of Pre-Hispanic Individuals Over Space and Time (A) Map with the locations of 86 ancient individuals (3 from our study are not included here due to very low coverage). Dotted lines represent regions defined for this study. Highland individuals are triangles and Coast individuals are circles. Coloring corresponds to genetic profiles, which in most cases match the geographic regions. (B) Groupings of ancient individuals based on geography and archaeological period (Table S1). Italics indicate previously published individuals, and sample sizes are in parentheses (yellow indicates shotgun sequences). Map was made with Natural Earth.

**Figure S1:**
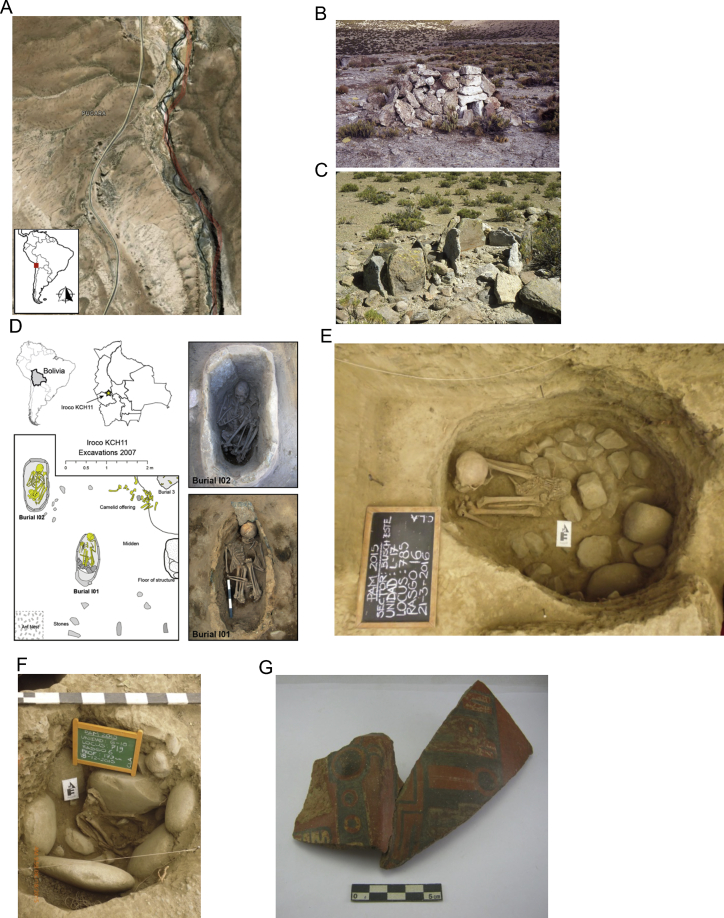
Images of Archaeological Sites, Related to STAR Methods (A) Location of Pukara in the Altiplano of northern Chile and South America (https://satellites.pro/mapa_de_Chile#-17.801972,-69.317661,19). (B) Undisturbed stone funerary cist of Pukara 3, close to the border of the cliff above Pukara 1. (C) Cist 1 and 2 (left to right) of Pukara 6. Disturbed and badly preserved human remains for this study come from cist or Tomb 1 (60 cm height, less than one m in diameter). The cists are surrounded by *tola* vegetation. (D) Iroco site (Bolivia). (E) Tiwanaku burial (Locus 785). (F) Tiwanaku cist tomb (Locus 719) with the sample individual 5 (MIS5). (G) Fragment of keru with the representation of the “staffed god.”

We assembled genome-wide data from 89 individuals from the Central and South-Central Andes over the past ∼9,000 years, including 65 newly reported individuals, and added data from an ∼1,600 BP individual from the Argentine Pampas region (Figures 1A, 1B, and S1; Table S1). We also report 39 direct radiocarbon dates (Table S1). The dataset includes individuals associated with a wide range of archaeological cultures from the Highlands and Coast of three geographic regions within present-day Peru: a northern zone we call “North Peru” (including sites in the Departments La Libertad and Ancash), a central zone we call “Central Peru” (Department of Lima), and a southern zone we call “South Peru” (including sites in the Departments of Ica, Ayacucho, Arequipa, Apurimac) spanning thousands of years through each of the regions. We also assembled data from Cusco in Peru, the South-Central Andean “Titicaca Basin” Highlands (spanning an ecologically and culturally unique region of southernmost Peru as well as western Bolivia), and “North Chile” (see Figure 1). Here, we use the term “archaeological cultures” as a proxy for the particular material cultures and site contexts from which our ancient individuals are derived, acknowledging that the actual human societies that produced the artifacts representing these material cultures often had substantially different social organizations, and our data are not sufficient to capture the full breadth and internal dynamics of each of them.

We combined the new data with previously published ancient DNA data from Lindo et al., 2018, Moreno-Mayar et al., 2018a, Moreno-Mayar et al., 2018b), Posth et al., 2018, Raghavan et al., 2015, Rasmussen et al., 2014, Rasmussen et al., 2015, Scheib et al., 2018, and Schroeder et al. (2018) and compared it with the genetic diversity of different present-day peoples (Barbieri et al., 2019, Mallick et al., 2016, Reich et al., 2012). We determined when the genetic structure observed today in the Central Andes first began to develop and assessed the degree to which gene flows over time have modulated this structure. Further, we investigated how changes in the population structure might correlate to archaeologically documented episodes of cultural, political, and socioeconomic change (summary of findings in Data S1).

### Ethics and Community Engagement

We acknowledge the Indigenous Peoples of Peru, Bolivia, Chile, and Argentina who supported this study as well as the ancient and present-day individuals whose samples we analyzed. The analysis of DNA from ancient individuals can have significant implications for present-day communities both because the studies can reveal how ancient people relate to present-day groups and also because the physical handling of the skeletal materials might be sensitive to the groups involved. Thus, it is important to engage with local communities and with scholars who work closely with these communities to incorporate these perspectives (Bardill et al., 2018, Claw et al., 2018) and to do so in a way appropriate for the particular Indigenous communities and political and social history in each region.

This study is the result of an international and inter-institutional collaborative effort that includes scientists from the countries where the ancient individuals originated. In all cases, the interest in genetic investigation of human remains centrally involved local co-authors, in most cases the archaeologists that excavated the sites. In many of these countries, archaeological investigations, as well as the permission to conduct biomolecular research on archaeological skeletal remains, is governed by national regulations. In Peru, for example, this is addressed in Ley General del Patrimonio Cultural de la Nación (Law No. 28296) (see also Herrera, 2011, Silverman, 2006). Our primary approach was thereby by necessity to consult with the provincial and state-based offices of the responsible institutions to obtain permission for analysis. In addition to this, however, we engaged with local communities throughout the study as detailed below.

All but one of the sample sets presented here were exported from their country of origin for this analysis and studied with direct permission of the local government. For example, the great majority of the samples newly reported in this study come from Peru, where this study was approved by the Ministry of Culture of Peru, which was originally created to revalue indigenous culture, past and present, to promote interculturality, and to fight against racism. The only exception is the San Sebastian samples (Cusco, Peru) that were part of a US collection and were studied there as part of a repatriation effort with permission of Peruvian institutions and are now curated in Cusco. Some of the samples, especially from coastal Peru, come from looted cemetery contexts, and the genomic data and direct radiocarbon dates generated here help to confirm their assignation to cultural epochs. Thus, this work helps to re-contextualize the individuals and has the potential to provide local communities with new ways to engage the past at disturbed sites.

For the individual from Argentina, in addition to obtaining permits from the provincial heritage institutions, the Indigenous community living near the site (Comunidad Indígena Mapuche-Tehuelche Cacique Pincen) approved the study after consultation and participation in the rescue excavation (the skeletal remains will be re-buried). The results of this and prior studies and their implications have been discussed with the community, and they have indicated support for this research in discussion with co-authors of the study. The regulations in Bolivia require archaeologists to consult with local communities before field research and turn in their research field reports to these communities. For the individuals from Chile, we obtained permits from the local heritage institutions, but no local Indigenous community lived near the site or indicated a connection to the analyzed skeletons.

Both before and during this study, there was substantial engagement with local communities by co-authors J.R.S., R.F., C.B., and G.P., who have a long-term commitment to specific regions and years of experience collecting data and returning results to the communities. Several of the co-authors presented the outcomes of this study and related archaeological and paleogenetic studies in the form of publicly accessible talks.

Data S1 is a translation of the Summary and Key Findings sections into Spanish to increase accessibility for non-English speakers, following the precedent established and used by the journal *Latin American Antiquity*.

## Results and Discussion

### Authenticity of Ancient DNA and Single Locus Patterns

**Figure S2:**
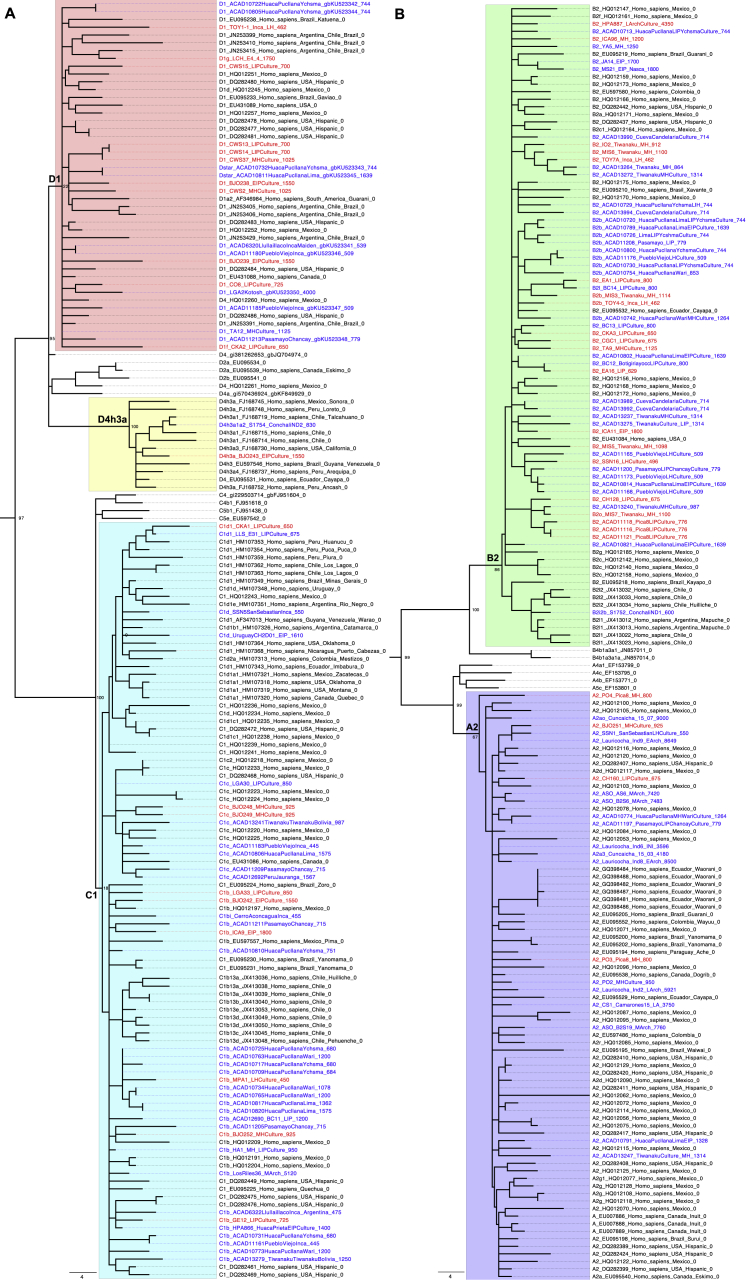
Mitochondrial DNA Tree, Related to Figures 3 and 4 Phylogenetic tree built with maximum parsimony algorithm of 136 ancient mtDNA (newly reconstructed sequences in red and previously published sequences in blue) and 197 modern mtDNA sequences (in black font) built using MEGA6 (Tamura et al., 2013) with an African mtDNA sequence used to root the tree (not shown). (A) Tree section including mtDNA haplogroups C1, D4h3a and D1. (B) Tree section including mtDNA haplogroup A2 and B2. Related to Table S1.

We evaluated the authenticity of the data based on: (1) characteristic cytosine-to-thymine substitution rate at the ends of the sequenced fragments from partially uracil-DNA-gylcosylase (UDG)-treated libraries of over 3% (Rohland et al., 2015); (2) point estimates of contamination in mtDNA below 5% (Furtwängler et al., 2018, Renaud et al., 2015); (3) point estimates of X chromosome contamination below 3% (only possible in males) (Korneliussen et al., 2014); and (4) point estimates of genome-wide contamination below 5% based on a method that leverages breakdown in linkage disequilibrium due to contamination (Nakatsuka et al., 2020). Individuals I1400, I01, and MIS6 were removed based on these analyses (full metrics are in Table S1). All common South American mtDNA haplogroups A2, B2a, B2b, C1b, C1c, D1, and D4h3a were represented (Figure S2), likely reflecting persistently large population sizes in the Central Andes (Harris et al., 2018, Llamas et al., 2016).

### Population Structure Has Early Holocene Roots

**Figure S3:**
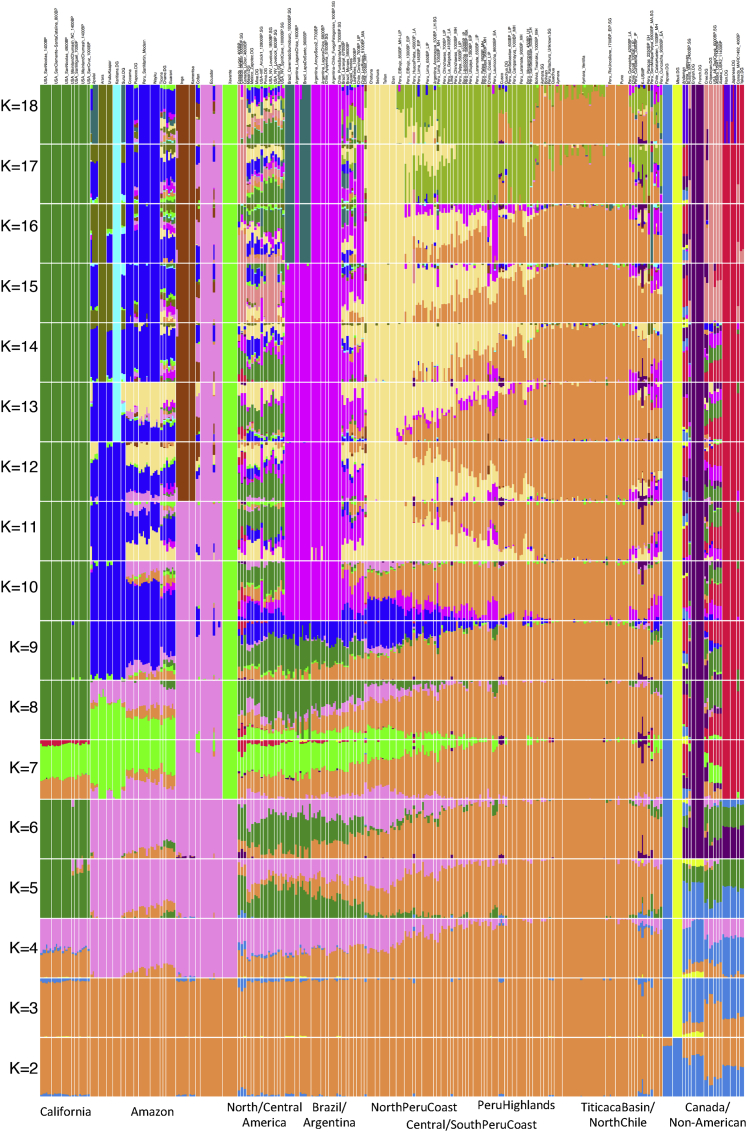
ADMIXTURE Plot at Different K Values, Related to Figure 2 The run with the highest log-likelihood score after 100 trials was taken for each K value.

**Figure 2:**
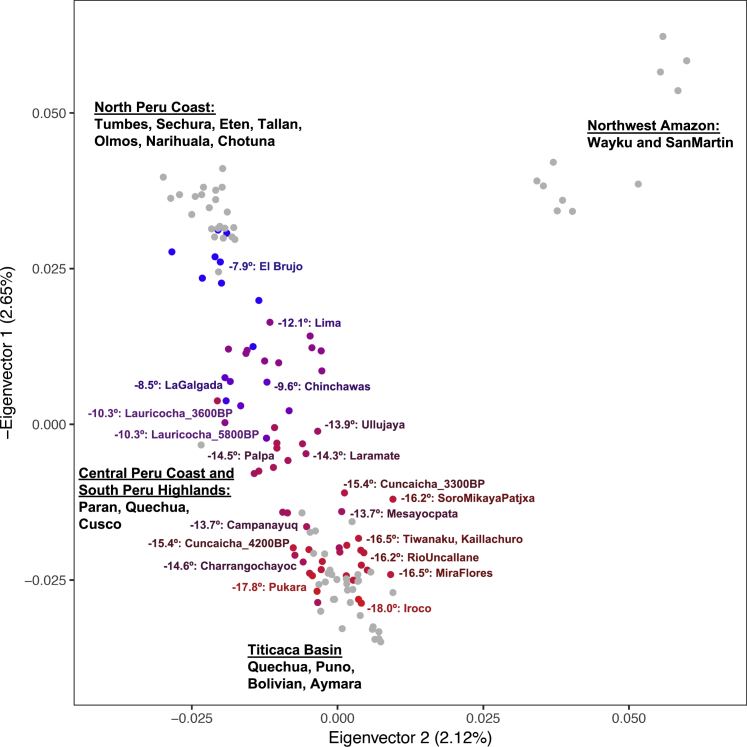
Principal Components Analysis (PCA) of Ancient Individuals Projected onto Modern Variation from Labeled Groups Modern individuals are in gray, and ancient individuals form a gradient that correlates to latitude (coloring is directly based on latitude with blue most north and red most south; numbers are latitude degrees). We removed 16 outliers from North Chile, Cusco, and Argentina that have evidence of ancestry from gene flows outside each region, and *Peru_Lauricocha_8600BP* and *Peru_Cuncaicha_9000BP*, which were too old to share the latitudinal cline (Figure S4 includes them). The percentage of total variation explained by each PC is shown in parentheses on each axis.

**Figure S4:**
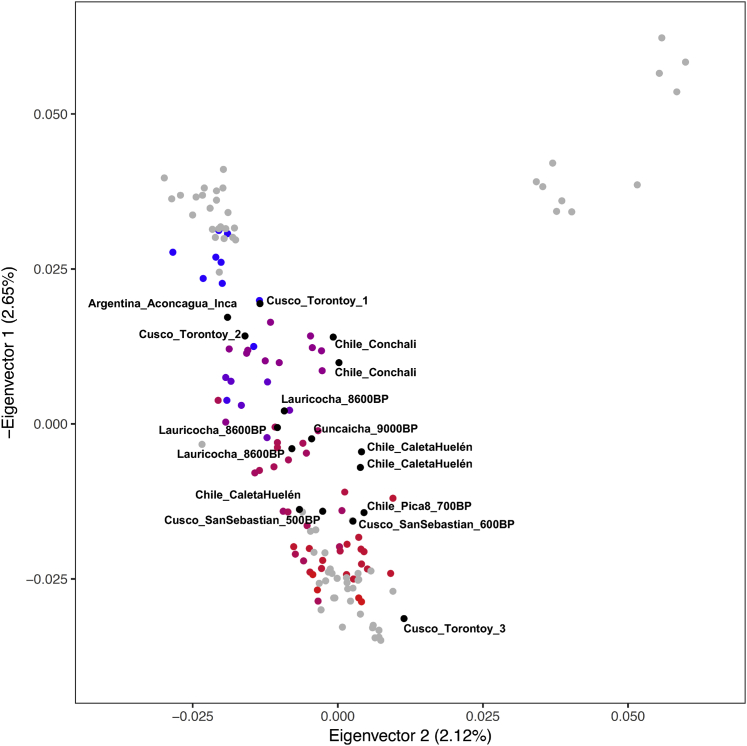
PCA of Additional Samples Removed from Figure 2, Related to Figure 2 Principal Components Analysis (PCA) of ancient samples projected onto modern individuals from the Andes and Amazon region (Barbieri et al., 2019). Eigenvector 1 coordinates were reversed so that the PCA correlates with geographical structure. Ancient individuals of Figure 2 are shown on a blue-red latitudinal gradient. Modern individuals are in gray. In black are all individuals that we removed from Figure 2 due to their outlier status reflecting distinctive histories that we discuss in the text (*NorthChile* individuals, Cusco individuals, the Argentinian Inca individual, and the *Peru_Lauricocha_8600BP* and *Peru_Cuncaicha_9000BP* individuals). The percentage of total variation explained by each PC is shown in parentheses on each axis.

**Figure 3:**
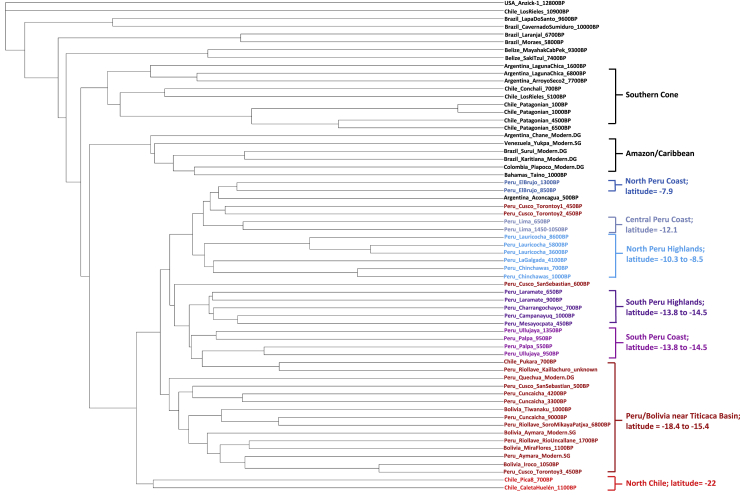
Neighbor-Joining Tree Based on Inverted Outgroup-*f*_*3*_ Statistics (1/*f*_*3*_(*Mbuti*; *Group1*, *Group2*)) Only individuals with >40,000 SNPs are included.

**Figure S5:**
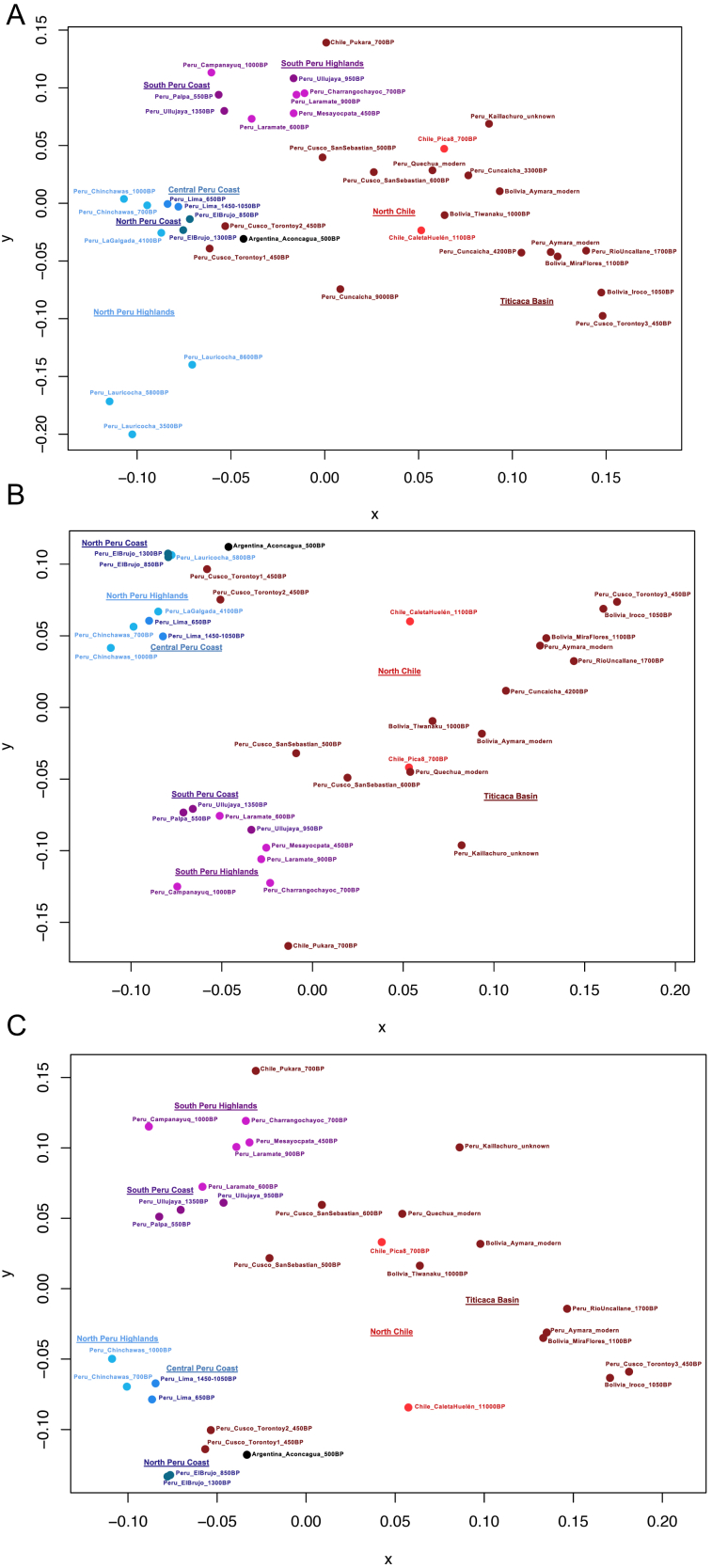
Multi-Dimensional Scaling (MDS) Plot of the Matrix of Statistics of the Form 1-*f*_*3*_(*Mbuti*; *South American 1*, *South American 2*), Related to Figure 2 Only individuals with > 100,000 SNPs are included. (A) Plot of all groups. (B) Plot of all groups younger than ~5900 BP, with the outliers *Peru_Lauricocha_3600BP* and *Peru_Cuncaicha_3300BP* removed. (C) Plot of all Andean groups younger than ~3000 BP.

Restricting to autosomal data, we performed a qualitative assessment of the population structure using unsupervised ADMIXTURE (Figure S3) and principal-component analysis (PCA) (Figures 2 and S4). We also generated a neighbor-joining tree and multi-dimensional scaling (MDS) plots of the matrix of “outgroup-*f*_*3*_” statistics of the form *f*_*3*_(*Mbuti*; *Pop1*, *Pop2*), which measure shared genetic drift between population pairs (Figures 3 and S5). Genetic structure strongly correlates with geography since at least ∼2,000 BP, with the first eigenvector in PCA corresponding to a north-to-south cline and the second separating Northwest Amazon groups from Central and South-Central Andean groups (Figure 2). The genetic structure is consistent with patterns expected from isolation-by-distance or gene flow among neighbors, with geographically closer individuals sharing more alleles than people separated by long distances (Figure 3). The oldest individuals (*Peru_NorthHighlands_Lauricocha_8600BP* and *Peru_SouthHighlands_Cuncaicha_9000BP*) did not plot in a position corresponding to their location, as expected, because these individuals were not affected by the shared drift and gene flow among geographic neighbors that shaped the population structure of much more recent individuals (Figure S4).

We examined how genetic structure evolved over time using statistics of the form *f*_*4*_(*Mbuti*, *Test*; *Pop1*, *Pop2*) where *Pop1* and *Pop2* were groups of similar time period, iterating over all other populations in our dataset as *Test*. We created analysis clusters for the regions labeled in Figure 1 (*NorthPeruHighlands*, *NorthPeruCoast*, *CentralPeruCoast*, *SouthPeruHighlands*, *SouthPeruCoast*, and *NorthChile)* where all ancient individuals younger than ∼2,000 BP were grouped (the Cusco individuals were excluded from *SouthPeruHighlands* for reasons discussed below) based on our empirical finding of a high degree of genetic homogeneity in each region since ∼2,000 BP (see below).

We first computed statistics of the form *f*_*4*_(*Mbuti*, *Test*; *Coast*, *Highlands*), declaring significance if the statistics were more than 3 standard errors from zero. *Test* individuals that share significantly more alleles with either Coast or Highlands groups must have lived when population structure existed that distinguished the Highlands regions of the Central and South-Central Andes from other parts of South America and thus provide a minimum on the date of the structure.

The North Peru Highlands reveal substantial continuity over seven millennia as shown by excess allele sharing of *Peru_NorthHighlands_Lauricocha_8600BP* to *NorthPeruHighlands* relative to *NorthPeruCoast* (Table S2A). Similarly, in the South Peru Highlands, *Peru_SouthHighlands_Cuncaicha_4200BP* shares more alleles with *SouthPeruHighlands* than with *SouthPeruCoast*. Thus, the oldest Highlands individuals were from populations that contributed more to later Highlands than to Coast groups, suggesting that the distinctive ancestry of late Highlands groups was already beginning to be established in the Highlands many thousands of years before. The long-standing genetic distinctiveness of the Highlands and Coast peoples is consistent with archaeological evidence that inhabitants of the Coast and the Highlands often relied on different subsistence strategies and had very different mobility patterns for millennia (Aldenfelder, 2008, Capriles et al., 2016b, Silverman, 2004). A better understanding of the distinctive ancient lineages that we detect in the Coast groups will require older genomes from the Coast.

A minimum date of development of North-South substructure can be inferred from the age of the earliest pair of North versus South groups that show asymmetric relationships with later individuals from the North and South. The earliest Peruvian Highlands individuals were symmetrically related to post-2,000 BP individuals (except for a degree of local continuity over ∼5,000 years in the Lauricocha site), but structure is evident by ∼4,200 BP, because *NorthPeruCoast*, *CentralPeruCoast*, and *NorthPeruHighlands* had significant affinity for *Peru_NorthHighlands_Lauricocha_5800BP* or *Peru_NorthHighlands_LaGalgada_4100BP* relative to *Peru_SouthHighlands_Cuncaicha_4200BP*, which instead had significant affinity for *NorthChile* and Titicaca Basin groups (Table S2B). The northern and southern lineages must have split at least ∼5,800 BP (the date of *Peru_NorthHighlands_Lauricocha_5800BP*), although we can only be confident that a north/south structure that correlates with the post-2,000 BP structure was established by the date of *Peru_SouthHighlands_Cuncaicha_4200BP*. This roughly corresponds to the onset of the Late Preceramic Period (∼5,000 BP), when increasing economic, political, and religious differentiation between Central Andean regions becomes evident archaeologically, and when levels of mobility decreased at the Coast and slightly later in the Highlands and Altiplano (Aldenfelder, 2008, Pozorski and Pozorski, 2008, Quilter, 2013). This occurred in tandem with increasing reliance on plant cultivation (Arriaza et al., 2008, Dillehay et al., 2007, Hastorf, 2008, Quilter, 2013, Rick, 1988, Rivera, 1995), which has been hypothesized to have contributed to rapid population growth in some regions (Gayo et al., 2015, Goldberg et al., 2016, Gómez-Carballa et al., 2018). A greater reliance on plant cultivation documented in the archaeological record from this period could plausibly contribute to increased sedentism and reduced gene flow, potentially contributing to the North-South substructure we observe beginning to develop by this period. However, it is important to note that demographic changes in the Andes most likely had various tempos and sequences in different regions; thus, data from larger samples sizes from this region around this time are necessary to gain greater clarity on this development of substructure.

### Gene Flow after the Establishment of Population Structure

**Figure S6:**
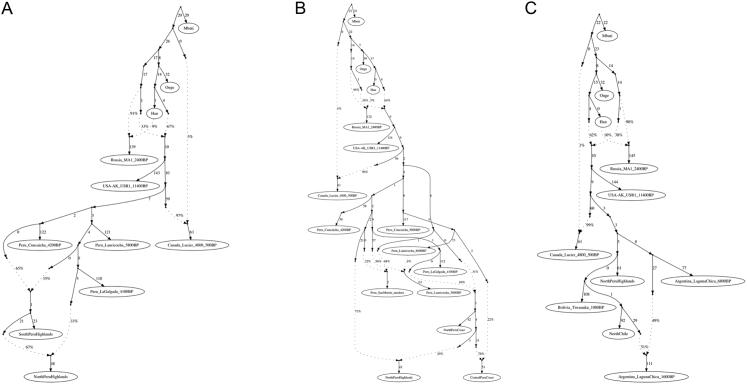
Admixture Graphs Generated through a Semi-Automated Process, Related to Figures 3 and 4 and Table S1 (A) This is the best fitting graph relating these groups (maximum |Z-score| = 2.5). Mixtures between South Peru Highlands (*Peru_Cuncaicha_4200BP*) and North Peru Highlands (*Peru_Lauricocha_5800BP* and *Peru_LaGalgada_4100BP*) were required in all fitting graphs (maximum |Z| < 3). (B) Admixture graph modeling Amazonian ancestry (related to *Peru_SanMartin_modern*) in *NorthPeruCoast*, *NorthPeruHighlands*, and *CentralPeruCoast*. Maximum |Z-score| = 3.0. (C) Admixture graph that fits the data (maximum |Z-score| = 3.1), showing *Argentina_LagunaChica_1600BP* as admixed from a population related to *Argentina_LagunaChica_6800BP* and *NorthChile*.

We document gene flow between the North and South Peru Highlands after the establishment of initial population structure through significantly more allele sharing of *SouthPeruHighlands* with *NorthPeruHighlands* than with *Peru_NorthHighlands_Lauricocha_5800BP* (Table S3A). We fit an admixture graph (Patterson et al., 2012) (Figure S6A) by systematically searching through all graphs with three or fewer admixture events among ancient Native Americans. *SouthPeruHighlands* could only fit as a mixture between groups related to *Peru_SouthHighlands_Cuncaicha_4200BP* and *Peru_NorthHighlands_Lauricocha_5800BP*. This could reflect gene flow between the regions and/or a mixture from a third unsampled population that affected both regions. We could not determine the directionality of gene flow due to a lack of very ancient South Peru Highlands individuals (Cuncaicha is further south than our later South Peru Highlands series and has ancestry more consistent with later Titicaca Basin individuals). A speculative possibility is that this admixture relates to the archaeologically documented Chavin sphere of influence (Burger, 2019) that involved cultural interaction between the North Peru Highlands (Ancash) to at least the Ayacucho region (“*SouthPeruHighlands*” in this study) ∼2,900–2,350 BP as reflected in the exchange of goods like cinnabar and obsidian, and by a widespread shared material culture style manifest across the Central Andes between Jaen in the north and Ayacucho in the south and along the north-central Pacific coast (Burger, 2008, Burger, 2019, Matsumoto et al., 2018). This scenario does not imply that the gene flow must have originated from Chavin, but that increased cultural and material exchange between the regions was accompanied by gene flow in one or both directions, although future work is necessary to test this hypothesis.

We also document gene flow between the Highlands and the Coast in North Peru based on significantly more allele sharing of *NorthPeruHighlands* with *NorthPeruCoast* than to *Peru_NorthHighlands_Lauricocha_5800BP* and of *CentralPeruCoast* with *Peru_NorthHighlands_Lauricocha_5800BP* relative to *Peru_NorthHighlands_Lauricocha_8600BP* (Fehren-Schmitz et al., 2014) (Tables S3A and S4). We detect gene flow connecting the Titicaca Basin to the South Peru Highlands and North Chile prior to ∼2,000 BP through significant allele sharing of *SouthPeruHighlands* and *NorthChile* with *Peru_TiticacaBasin_RioUncallane_1700BP* relative to *Peru_TiticacaBasin_SoroMikayaPatjxa_6800BP* and *Peru_TiticacaBasin_RioUncallane_1700BP* and *NorthChile* with *Peru_Cuncaicha_4200BP* relative to *Peru_Cuncaicha_9000BP*. This accords with archaeological evidence of cultural exchange prior to ∼2,000 BP between these regions (Olson et al., 2020, Santoro et al., 2017) as well as observations of gene flow between the regions based on mtDNA, although our date estimates precede the estimated dates from the mtDNA studies by ∼1,000 years (Aufderheide et al., 1994, Moraga et al., 2005, Rivera, 1991, Rothhammer et al., 2004).

### Continuity in Most Regions after ∼2,000 BP

After ∼2,000 BP, we observe genetic homogeneity within most regions to the limits of our statistical resolution. This is evident when we group individuals by geography, time period, and archaeological cultural context and compute statistics of the form *f*_*4*_(*Mbuti*, *Test*; *Pop1*, *Pop2*), where Pop1 and Pop2 are two groups within the same geographical/temporal/archaeological category, and *Test* is a range of other groups outside the region. Statistics in almost all regions were consistent with zero (Table S3B), indicating that the *Test* population shares alleles at approximately an equal rate with *Pop1* or *Pop2*. We also used *qpWave* (Reich et al., 2012) to agglomerate the *f*_*4*_-statistics for each (Pop1, Pop2) pair, computing a single p value that takes into account the correlation in ancestry among the *Test* populations used as outgroups (Table S5). The only exceptions to the evidence of homogeneity are in Cusco and the Titicaca Basin (see below); hence, we split post ∼2,000 BP individuals in these two regions into homogeneous analysis subgroups.

The persistent regional substructure we detect over the last two millennia is notable given the dynamic changes of archaeological cultures, territorial expansions, and ever-changing intercultural interactions. Within the span of the *NorthPeruCoast* time series at the site of El Brujo from ∼1,750–560 BP, the Moche (∼1,850–1,250 BP) developed and were succeeded by the Lambayeque (∼1,250–575 BP) (Castillo Butters and Uceda, 2008), yet we detect no significant difference in ancestry relative to individuals from outside the region. In the *NorthPeruHighlands* we find continuity in the Ancash region at Chinchawas and LaGalgada (∼1,200–550 BP). In the *CentralPeruCoast* time series we find continuity in the Lima region from ∼1,850–480 BP through the period of cultural influence of the Highland Wari polity (∼1,350–950 BP) (Isbell, 2008). In the *SouthPeruCoast*, we find continuity at Ica and Palpa from (∼1,480–515 BP), spanning the demise of the Nasca culture (∼2,050–1,200 BP). In the *SouthPeruHighlands* time series, we find continuity in the region including the Laramate Highlands, Mesayocpata, Charangochayoc, and Campanayuq (∼1,150–390 BP) despite Wari influence. Thus, the peoples of each region in each period are consistent with having become the primary demographic substrate for those in the next, suggesting that cultural changes were largely driven by political/territorial restructuring with little evidence of large-scale mass migrations such as those that have been documented in some other regions of the world through ancient DNA (Valverde et al., 2016). This, of course, does not exclude the possibility of smaller scale movements of people, Elite-Dominance scenarios, or other dynamic demographic processes that left genetic signatures not detectable by our analysis. It is possible that phenomena of this type could be detected with large sample sizes that could reveal outlier individuals from some regions with different ancestries. However, our results add to the body of evidence consistent with conflict not having had a strong influence on demography over this period, most notably showing that the cultural impact of the Wari on coastal regions previously dominated by the Moche and Nasca was not mediated by large-scale population replacement or admixture (Castillo, 2003).

**Figure S7:**
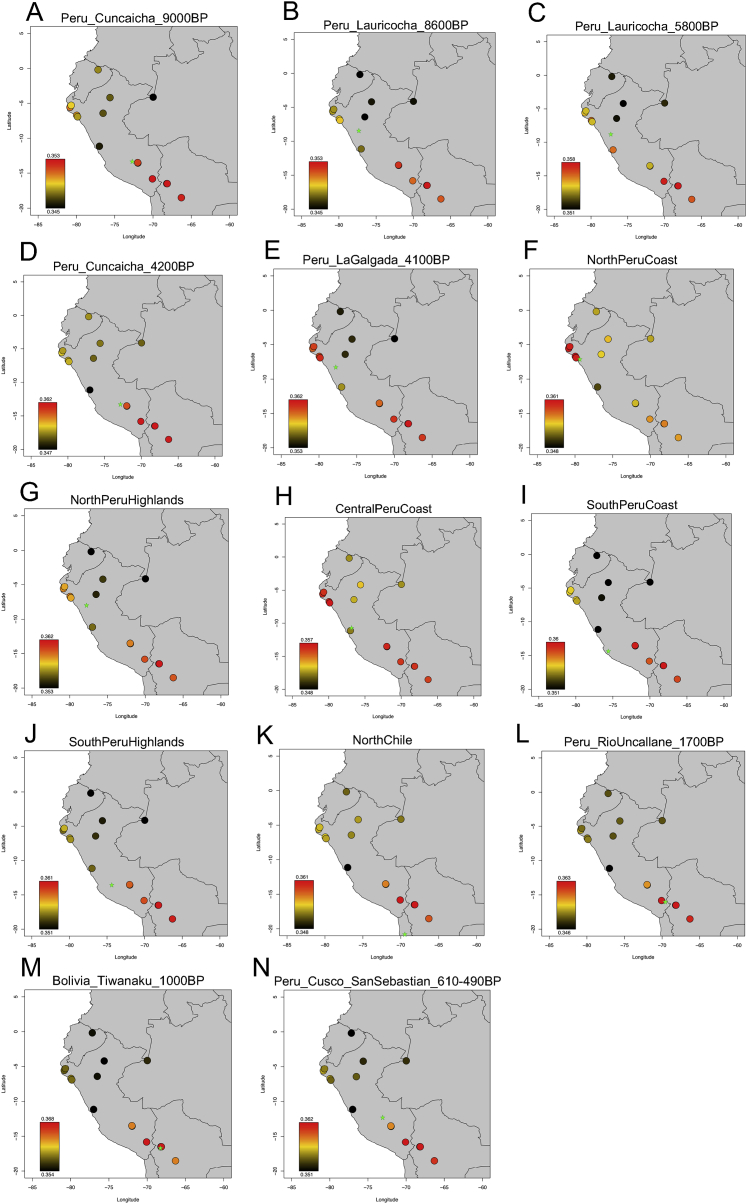
Heatmap of Outgroup *f*_*3*_-Statistics, Related to Figure 2 Color coding is based on statistics of the form f_3_(Mbuti; Ancient, Modern), where the Ancient groups are (A) Peru_SouthHighlands_Cuncaicha_9000BP, (B) Peru_NorthHighlands_Lauricocha_8600BP, (C) Peru_NorthHighlands_Lauricocha_5800BP, (D) Peru_SouthHighlands_Cuncaicha_4200BP, (E) Peru_NorthHighlands_LaGalgada_4100BP, (F) NorthPeruCoast, (G) NorthPeruHighlands, (H) CentralPeruCoast, (I) SouthPeruCoast, (J) SouthPeruHighlands, (K) NorthChile, (L) Peru_TiticacaBasin_RioUncallane_1700BP, (M) Bolivia_TiticacaBasin_Tiwanaku_1000BP, and (N) Peru_Cusco_SanSebastian_610-490BP. Modern groups are from Barbieri et al. (2019). Related to Table S6.

The genetic structure established in each region from ∼2,000–500 BP is strongly echoed in the genetic structure of present-day Indigenous peoples. This is evident in the outgroup-*f*_*3*_-based tree and MDS plots where ancient individuals cluster with modern individuals from the same region (Figures 3 and S5). In addition, when we computed statistics of the form *f*_*3*_(*Mbuti*; *Ancient Andean*, *Present-Day South American*) (Barbieri et al., 2019), we observe qualitatively that the present-day individuals are most closely related to the ancient individuals from their region (Figure S7), a finding that is significant as measured by *f*_*4*_-statistics (Table S6). For example, we observe excess allele sharing of the *NorthPeruCoast* individuals with Sechura (a present-day North Peru Coast group) compared to Puno (a present-day Titicaca Basin group). Thus, the forced migrations imposed by the Inca and Spanish in these regions did not completely disrupt the genetic population structure that existed prior to these events (Iannacone et al., 2011). Another example is significantly more allele sharing of *CentralPeruCoast* with Quechua speakers relative to Aymara speakers from the same region (Reich et al., 2012), and, conversely, significantly more allele sharing of *NorthChile* and Titicaca Basin groups with Aymara speakers relative to Quechua speakers. This correlates to the geographic range of speakers of these two languages found today, with Aymara more circumscribed to the shores of the Titicaca Basin and southern territories, and Quechua in the north (Heggarty, 2008) (Table S6). We emphasize that this is a statement about genetic continuity, not a connection to speakers of specific languages: due to our lack of data from ancient individuals known to speak Quechua or Aymara, we cannot determine whether the language distribution followed the same pattern of geographic continuity, especially because the modern distribution of Quechua and Aymara is strongly influenced by Spanish colonial politics, as well as post-colonial state marginalization of those languages.

### Cosmopolitanism during the Tiwanaku and Inca Periods

We document long-range mobility and genetic heterogeneity at the sites of Tiwanaku in the Titicaca Basin and Cusco associated with the administrative centers of the Tiwanaku polity (1,400–950 BP) and Inca Empire (∼550–420 BP), respectively and successively. At Tiwanaku, this is evident in significantly more allele sharing of *SouthPeruHighlands* with *Bolivia_Tiwanaku_1000BP* (individuals from Tiwanaku’s administrative center) than with all other Titicaca Basin groups from this period in the Tiwanaku sphere of influence (spanning North Chile, Western Bolivia, and South Peru) (Tables S4 and S7A). This could potentially be explained by the pull-factor that a major administrative, religious, and urban center like Tiwanaku (Isbell, 2008) had on individuals from surrounding groups. While what we call *SouthPeruHighlands* broadly falls into the sphere of influence of the Wari polity at the time of Tiwanaku, this does not seem to restrict such movement, which could be a sign of the limited impact the Wari polity had on some regions in their sphere of influence as suggested by some scholars (Jennings, 2010). After the Tiwanaku disintegration, but before the expansion of the Inca Empire, we observe two ∼700 BP individuals from close to the border of present-day Chile, Peru, and Bolivia that shared more alleles with *SouthPeruHighlands* than with Titicaca Basin groups, including even Tiwanaku (Figures 2 and 3; Tables S4 and S7A). These individuals were from a cemetery of herders and their ancestry could be reflective of migrants from the South Peru Highlands. The archaeological record indicates that the end of both Wari and Tiwanku led to a spread of camelid pastoralism, which involved increased regional mobility and could have led to the observed migration (Covey, 2008, Stanish, 2003).

**Figure 4:**
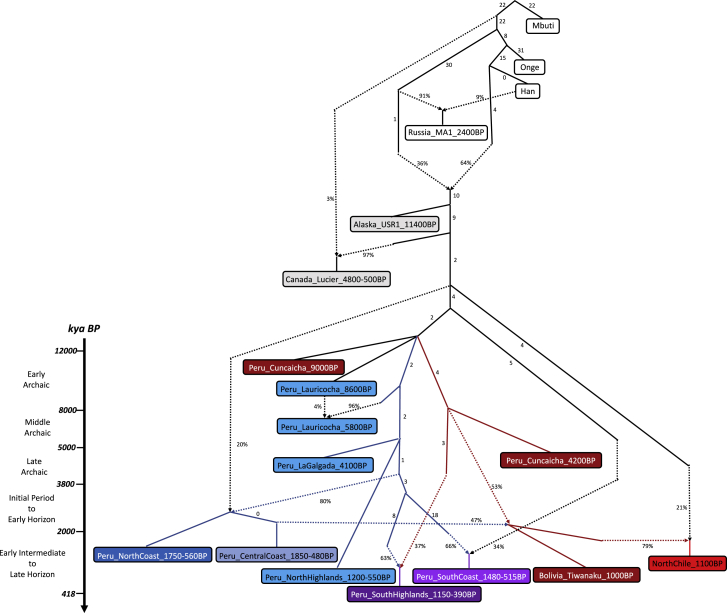
Overview of Findings Admixture graph fit (maximum |*Z*| score between observed and expected *f-*statistics is 3.1). *Chile_Pica8_700BP* was removed from *NorthChile* due to low coverage.

During the Inca Empire, we detect significant heterogeneity in individuals within the Cusco region (San Sebastian) and the Sacred Valley (Torontoy). This is seen in Figures 3, S3, and S4 where the individuals cluster with *NorthPeruCoast*, *SouthPeruHighlands*, and Titicaca Basin groups (Figure 4; Tables S4 and S7B). The pre-Hispanic Cusco individuals are less related to present-day Cusco individuals (Barbieri et al., 2019) than to groups outside the region (Tables S4 and S7B). Specifically, we find that relative to the ancient Cusco individuals from the San Sebastian or Torontoy sites, *SouthPeruHighlands* always shows significantly more allele sharing with present-day Cusco, with the signal maximized by the *Peru_Chanka_Charangochayoc_700BP* group, which originates from the site of the same name in the Lucanas province, Ayacucho Region, ∼300 km to the west of Cusco. The process that led to present-day peoples of Cusco harboring ancestry distinctive from the ancient Cusco individuals is an important topic for future research. Possible scenarios include policies by the Inca or Spanish to move groups into or out of that region (*mitma* forced relocation) or recent economic diasporas into the region (Alconini and Covey, 2018) (a large-scale rural exodus into urban areas was documented in the 19^th^ and 20^th^ century). These patterns could also be explained if the ancient Cusco individuals were immigrants or recent descendants of immigrants, as has been shown for burials at Machu Picchu employing morphological and isotopic data (Burger et al., 2003, Turner et al., 2009, Verano, 1912).

The dataset also highlights a case of extreme mobility during the Inca period. Published data from an Inca culture-associated boy found in the Southern Andes (Moreno-Mayar et al., 2018b) (*Argentina_Aconcagua_500BP*) is most closely related to *NorthPeruCoast* (Figure 3; Table S3C), reflecting long-distance movement of the child for his sacrifice (Gómez-Carballa et al., 2015, Llamas et al., 2016, Salas et al., 2018), likely from the same region as the two Inca period *NorthPeruCoast*-related *Cusco_Torontoy* individuals, as they form a clade with each other in *qpWave* analyses (Table S7B). This suggests that a particular site in the North Peru coastal region was likely important for the Inca (differing from prior reports that suggested the Inca sacrifice was from the Central Coast) (Gómez-Carballa et al., 2015, Llamas et al., 2016, Salas et al., 2018).

### Genetic Exchange between the Northwest Amazon and North Peru

We tested for gene flows between the Central Andes and other regions. We observe excess allele sharing between the Northwest Amazon and North Peru as shown by significantly more affinity of Indigenous peoples from the western Amazon (*SanMartin*, *Ticuna*, *Wayku*, and *Surui*) to *NorthPeruCoast*, *NorthPeruHighlands*, or *CentralPeruCoast* than to *SouthPeruCoast* or the earlier *Lauricocha_5800BP* individual (Table S7C). We used *qpAdm* to model *Peru_Amazon_SanMartin_Modern* as a mixture of 29% ancient North Highlands-related ancestry (related to *Peru_NorthHighlands_LaGalgada_4100BP*) and 71% Amazonian ancestry (related to *Brazil_Amazon_Karitiana_Modern*) (±11%, quoting one standard error), suggesting that at least some modern Northwest Amazonian groups harbor Andean-related ancestry. When we modeled Peruvian groups as a mixture of *Peru_WestAmazon_SanMartin_Modern* and *Peru_NorthHighlands_LaGalgada_4100BP*, we also identified coastal individuals with significantly non-zero Amazonian-related ancestry (e.g., 39% ± 14% in *CentralPeruCoast*) (Figure S6B; Table S7C), suggesting bi-directional gene flow with Amazonian-related ancestry affecting the Peruvian North and Central Coast more than the Highlands (Barbieri et al., 2014, Di Corcia et al., 2017, Gnecchi-Ruscone et al., 2019, Harris et al., 2018, Reich et al., 2012, Rothhammer et al., 2017). We detect no gene flow between the North Coast and Amazonian groups to the North, because we found no significant affinity of any modern Amazonian groups from Ecuador or Colombia to *NorthPeruCoast* relative to *Lauricocha_5800BP*.

The stronger signal of Amazonian-related ancestry in the North and Central Coast relative to the North Highlands suggests that gene flow could have occurred over the low mountain ranges of North Peru (Huancabamba deflection), rather than across the high-altitude mountain ranges that dominate the Andes further south and potentially are a larger barrier to gene flow, or if Highlands groups maintained high social barriers to admixture from the Amazonian groups. We used the software *DATES* (Narasimhan et al., 2019), which models allele covariance over genetic distance to measure admixture dates, and found that the admixtures occurred ∼1,478 ± 252 years ago in *NorthPeruCoast* and ∼1,153 ± 90 years ago in *CentralPeruCoast* (Table S7C), consistent with the hypothesis of a southward migration pattern.

We do not observe tropical lowlands-derived gene-flow into the Titicaca Basin or Northern Chile as reported in studies based on mitochondrial DNA (Rothhammer and Dillehay, 2009, Rothhammer et al., 2017). There is strong archaeological evidence for the exchange of food crops and other goods between the lowlands east of the Andes and the Chilean North coast (Rothhammer and Dillehay, 2009, Santoro, 1980), but it is possible this did not lead to gene flow detectable in the *NorthChile* individuals tested here, which post-date the postulated exchange by 2,000–3,000 years (Rothhammer and Dillehay, 2009). Because we do not have any DNA from ancient Amazonians, we cannot exclude gene flow from past groups carrying so-far undetected linages.

### Gene Flow between the Argentine Pampas and South-Central Andes

We detect significantly more allele sharing of *SouthPeruHighlands*, *SouthPeruCoast*, *CentralPeruCoast*, and Titicaca Basin groups to *Argentina_LagunaChica_1600BP* relative to *Argentina_LagunaChica_6800BP*. This likely reflects gene flow between the Pampas and the Central Andes, consistent with previous claims (Gómez-Carballa et al., 2018, Muzzio et al., 2018). Using *qpAdm,* we fit *Argentina_LagunaChica_1600BP* as a mixture of 80% ± 12% ancestry related to *Argentina_LagunaChica_6800BP* and 20% ± 12% ancestry related to a representative Andes group giving the lowest standard error (*CentralPeruCoast*). We also fit *CentralPeruCoast* as 77% ± 17% related to *Peru_Cuncaicha_4200BP* and 23% ± 17% related to *Argentina_LagunaChica_1600BP* (Figure S6C; Table S7D). Pottery and metal objects of South Andean origin are found in the Araucania region in the western Pampas dating to at least ∼1,000 BP (Berón, 2007), and skeletons from Chenque 1 in the Pampas have been suggested to have South Andean isotopic signatures (Berón et al., 2013). Taken together, there is thus compelling evidence for human movements as well as cultural interactions between these regions at least ∼1,600 BP.

### Distinctive Ancestry Profile that Arrived by ∼4,200 BP Fully Integrated by ∼2,000 BP

**Figure S8:**
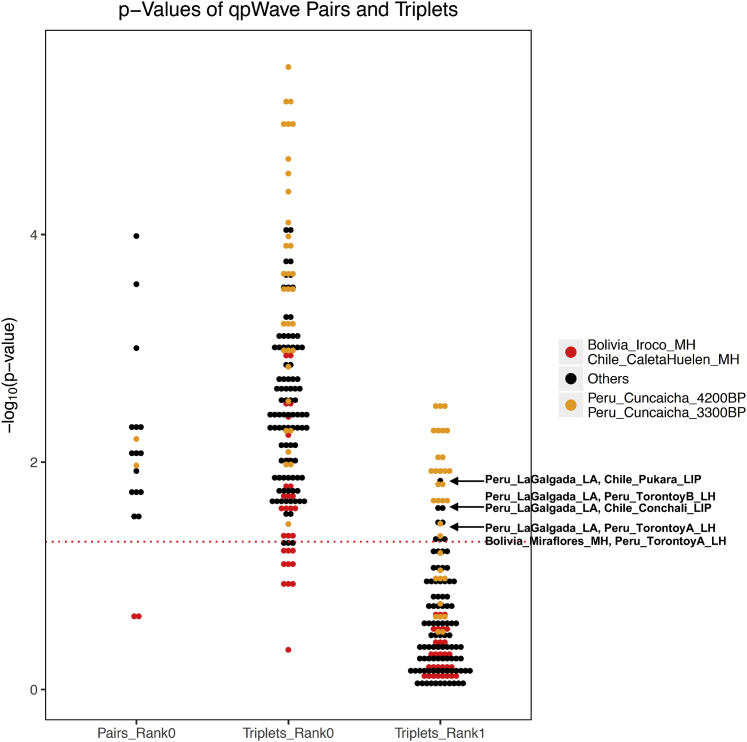
*qpWave* Analyses, Related to STAR Methods *qpWave* analyses with all pairs (Pairs_Rank0) or triplets (Triplets_Rank0 or Triplets_Rank1) of all Andean and North Chile groups when compared with *Peru_Lauricocha_8600BP*. Rank 0 and 1 refers to a model in which all populations in the analysis fit as derived from one or two ancestral populations, respectively, relative to the outgroups (rejection of these ranks means that additional sources of ancestry are required to model the populations). Modern groups and shotgun samples were excluded due to potential for artifacts. *Peru_Lauricocha_5800BP* and *Peru_Lauricocha_3600BP* were also excluded due to their previously known lack of California Channel Island-related ancestry (Posth et al., 2018). Color-coding refers to pairs or triples that include groups that consistently do not fit in some two-source or three-source models: the Cuncaicha 3300 BP and 4200 BP individuals are the only groups that are consistently poorly modeled even without a third source of ancestry. Related to Table S8.

A previous study (Posth et al., 2018) detected a signal of differential North American-relatedness in groups from Southern Peru and North Chile after ∼4,200 BP relative to earlier groups. We used our data to explore the timing and geographic extent of the spread of this ancestry, using the same approach as the previous work on this topic (Posth et al., 2018). All of the groups after ∼4,200 BP, except for the Lauricocha individuals, *Chile_CaletaHuelen_1100BP*, and *Bolivia_Iroco_1050BP* were significant for two sources of ancestry (p < 0.05) (Figure S8; Table S8), suggesting that the California Channel Island-related ancestry spread throughout all of the Andes by at least ∼2,000 BP. With the software DATES, we measured the admixture time to be ∼5,000 ± 1,500 years ago (Table S8).

### Summary Model and Conclusions

**Figure 5:**
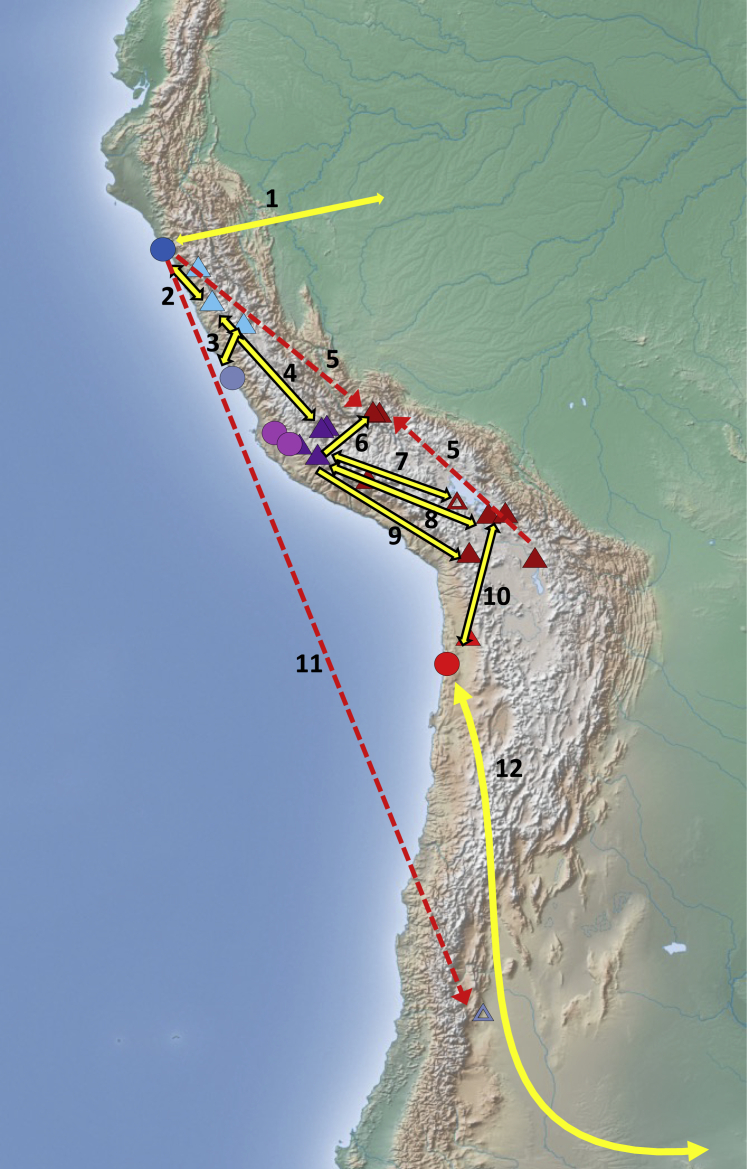
Map Summarizing Genetic Exchanges in the Central Andes (1) Bi-directional mixture between the North and Central Coasts and the Northwest Amazon. (2) Genetic exchange between *NorthPeruCoast* and *NorthPeruHighlands*. (3) Genetic interaction between *CentralPeruCoast* and *NorthPeruHighlands_Lauricocha* before ~5800 BP. (4) Genetic exchange between *NorthPeruHighlands* and *SouthPeruHighlands*. (5) Individuals of *NorthPeruCoast* and Titicaca Basin-related ancestry found in Cusco (Torontoy) during the Inca Empire (~450 BP). (6) Spread of *SouthPeruHighlands-*related ancestry into the Cusco region 450 BP–present. (7) Genetic exchange between *SouthPeruHighlands* and Titicaca Basin before 1,700 BP. (8) Greater allele sharing between Tiwanaku and *SouthPeruHighlands* relative to other individuals in Titicaca Basin during the Tiwanaku period (~1,000 BP). (9) *SouthPeruHighlands*-related ancestry found in Pukara in Northern Chile ~700 BP. (10) Genetic exchange between *NorthChile* and Titicaca Basin before ~1,700 BP. (11) *NorthPeruCoast*-related ancestry found in an Inca sacrifice victim in Argentina. (12) Gene flow between *NorthChile* or *SouthPeruHighlands* and the Pampas region of Argentina.

We used a semi-automated procedure to build an admixture graph to model representative ancient Central and South-Central Andeans (Patterson et al., 2012) (Figures 4 and 5). Our best fit recapitulates key findings from this study. The earliest Peruvians do not share genetic drift with the later groups in our dataset, except for local continuity at the Lauricocha site. The differentiation between North and South Peru Highlands correlating to later structure is only evident by 5,800–4,100 BP. Post ∼2,000 BP South Peru Highlands individuals are modeled as a mixture of earlier South Highlands and North Highlands-related ancestry. Deep ancestry is inferred in Coast individuals, while North Chile individuals can only be fit with ancestry from a different basal lineage. Post ∼2,000 BP individuals from the socio-political center of Tiwanaku exhibit mixtures of ancestry related to contemporary people from the Central Peru Coast and South Peru Highlands. An important direction for future work is to obtain ancient DNA from the Coast prior to ∼1,600 BP, as well as equally rich ancient DNA data from regions to the north, west, and south of the Central Andes, which will provide further important insights.

## STAR★Methods

### Key Resources Table

**Table:** 

REAGENT or RESOURCE	SOURCE	IDENTIFIER
**Chemicals, Peptides, and Recombinant Proteins**		

Pfu Turbo Cx Hotstart DNA Polymerase	Agilent Technologies	600412
Herculase II Fusion DNA Polymerase	Agilent Technologies	600679
2x HI-RPM hybridization buffer	Agilent Technologies	5190-0403
0.5 M EDTA pH 8.0	BioExpress	E177
Sera-Mag Magnetic Speed-beads Carboxylate-Modified (1 μm, 3EDAC/PA5)	GE LifeScience	65152105050250
USER enzyme	New England Biolabs	M5505
UGI	New England Biolabs	M0281
Bst DNA Polymerase2.0, large frag.	New England Biolabs	M0537
PE buffer concentrate	QIAGEN	19065
Proteinase K	Sigma Aldrich	P6556
Guanidine hydrochloride	Sigma Aldrich	G3272
3M Sodium Acetate (pH 5.2)	Sigma Aldrich	S7899
Water	Sigma Aldrich	W4502
Tween-20	Sigma Aldrich	P9416
Isopropanol	Sigma Aldrich	650447
Ethanol	Sigma Aldrich	E7023
5M NaCl	Sigma Aldrich	S5150
1M NaOH	Sigma Aldrich	71463
20% SDS	Sigma Aldrich	5030
PEG-8000	Sigma Aldrich	89510
1 M Tris-HCl pH 8.0	Sigma Aldrich	AM9856
dNTP Mix	Thermo Fisher Scientific	R1121
ATP	Thermo Fisher Scientific	R0441
10x Buffer Tango	Thermo Fisher Scientific	BY5
T4 Polynucleotide Kinase	Thermo Fisher Scientific	EK0032
T4 DNA Polymerase	Thermo Fisher Scientific	EP0062
T4 DNA Ligase	Thermo Fisher Scientific	EL0011
Maxima SYBR Green kit	Thermo Fisher Scientific	K0251
50x Denhardt’s solution	Thermo Fisher Scientific	750018
SSC Buffer (20x)	Thermo Fisher Scientific	AM9770
GeneAmp 10x PCR Gold Buffer	Thermo Fisher Scientific	4379874
Dynabeads MyOne Streptavidin T1	Thermo Fisher Scientific	65602
Salmon sperm DNA	Thermo Fisher Scientific	15632-011
Human Cot-I DNA	Thermo Fisher Scientific	15279011
DyNAmo HS SYBR Green qPCR Kit	Thermo Fisher Scientific	F410L
Methanol, certified ACS	VWR	EM-MX0485-3
Acetone, certified ACS	VWR	BDH1101-4LP
Dichloromethane, certified ACS	VWR	EMD-DX0835-3
Hydrochloric acid, 6N, 0.5N & 0.01N	VWR	EMD-HX0603-3

**Critical Commercial Assays**		

High Pure Extender from Viral Nucleic Acid Large Volume Kit	Roche	5114403001
MinElute PCR Purification Kit	QIAGEN	28006
NextSeq® 500/550 High Output Kit v2 (150 cycles)	Illumina	FC-404-2002
HiSeq® 4000 SBS Kit (50/75 cycles)	Illumina	FC-410-1001/2

**Software and Algorithms**		

Samtools	Li, 2011, Li et al., 2009	http://samtools.sourceforge.net/
BWA	Li and Durbin, 2009	http://bio-bwa.sourceforge.net/
ADMIXTOOLS	Patterson et al., 2012	https://github.com/DReichLab/AdmixTools
SeqPrep	https://github.com/jstjohn/SeqPrep	https://github.com/jstjohn/SeqPrep
bamrmdup	https://bitbucket.org/ustenzel/biohazard	https://bitbucket.org/ustenzel/biohazard
AdapterRemovalv2	Schubert et al., 2016	https://github.com/MikkelSchubert/adapterremoval
Dedeup	Peltzer et al., 2016	https://eager.readthedocs.io/en/latest/
smartpca	Patterson et al., 2006	https://www.hsph.harvard.edu/alkes-price/software/
ADMIXTURE	Alexander et al., 2009	https://www.genetics.ucla.edu/software/admixture/download.html
PMDtools	Skoglund et al., 2014	https://github.com/pontussk/PMDtools
Haplofind 2	Vianello et al., 2013	https://haplofind.unibo.it
Haplogrep	Kloss-Brandstätter et al., 2011, Weissensteiner et al., 2016	https://haplogrep.uibk.ac.at/index.html
Yfitter	https://sourceforge.net/projects/yfitter/	https://sourceforge.net/projects/yfitter/
ContamMix	Fu et al., 2013	https://github.com/DReichLab/ADNA-Tools
ANGSD	Korneliussen et al., 2014	https://github.com/ANGSD/angsd
MEGA6	Tamura et al., 2013	https://www.megasoftware.net
mapDamage2.0	Jónsson et al., 2013	https://ginolhac.github.io/mapDamage/
Geneious	https://www.geneious.com/	https://www.geneious.com/
MUSCLE	Edgar, 2004	https://www.drive5.com/muscle/
FigTree	http://tree.bio.ed.ac.uk/software/	http://tree.bio.ed.ac.uk/software/
PLINK2	Chang et al., 2015	https://www.cog-genomics.org/plink/2.0/
ContamLD	Nakatsuka et al., 2020	https://github.com/nathan-nakatsuka/ContamLD

**Deposited Data**		

Sequencing Data	European Nucleotide Archive	PRJEB37446
Genotype Data	Reich Lab website	https://reich.hms.harvard.edu/datasets

### Lead Contact and Materials Availability

#### Lead Contact

Further information and requests for resources and reagents should be directed to and will be fulfilled by the Lead Contact, Lars Fehren-Schmitz (lfehrens@ucsc.edu).

#### Materials Availability

This study did not generate new unique reagents.

### Experimental Model and Subject Details

#### Archaeological site information:

We generated new genome-wide data from skeletal remains of 66 ancient individuals:

Caleta Huelen 12, Chile: 3
Pukara, Chile: 2
Iroco, Oruro, Bolivia: 2
Miraflores, La Paz, Bolivia: 4
Tiwanaku, La Paz, Bolivia: 4
Monte Grande, Peru: 1
Los Molinos, Palpa, Peru: 2
Laramate, Peru: 4
Charangochayoc, Peru: 1
Ullujaya, lower Ica Valley, Peru: 3
Mesayocpata, Peru: 1
Torontoy, Cusco, Peru: 3
Campanayuq Rumi, Peru: 3
San Sebastian, Cusco, Peru: 3
Huaca Pucllana, Lima: 12
Chinchawas, Peru: 5
El Brujo, Peru: 9
La Galgada, Peru: 1
Pampas, Laguna Chica, Argentina: 1
Paracas, Peru 1
Huaca Prieta 1

#### Brief description of Archaeological Sites:

##### Caleta Huelén 12 (Chile): 1350-680 BP

• I2538: 900-680 calBP (1220 ± 20 BP, PSUAMS-1619)
• I2539: 1155-835 calBP (1320 ± 20 BP, PSUAMS-1619)
• I2540: 1350-1150 BP

The Caleta Huelén 12 individuals derive from a burial site associated with a campsite located on a marine terrace just south of the Rio Loa mouth in the Northern Chile Atacama Desert. Over 50 burials, mostly collective, have been studied in the past containing predominantly San Miguel type of ceramics (Late Intermediate Period, ∼1000 BP), but also other ceramic indicators such as Dupont, Taltape and wares especially related to Pica 8. The archaeological context revealed people with a maritime economy and very limited agricultural practice.

##### Pukara 6, Tomb 1 (Chile): 950-420 BP

• I14009: 795-690 calBP (890 ± 20 BP, PSUAMS-6819)
• I17497: 950-420 BP

The Pukara 6 site (Figure S1A) corresponds to a cemetery located in North Chile. Located on a hill on the western margin of the Caquena river (which acts as a border between Bolivia and Chile, ca. 4,000masl), Pukara-6 is situated in a typical Andean Altiplano steppe environment.

This landscape, with a low mean annual temperature (0-5°C) and an average of no more than 300 mm of precipitation, is known as puna or tolar. The vegetation, dominated by shrubs (locally known as tola) and perennial grasses (pajonal), is ideal for camelid pastoral economy, but does not permit agriculture (too high and cold). The Caquena river and the prairies generate basic conditions for human settling. Humans colonized the area by the end of the Pleistocene, and it is to this day, the home of several modern Aymara herding hamlets like Pukara. For more information see Díaz et al. (2019).

A total of 12 archaeological locations were inventoried numerated as follows: (1) Pukara 1 a rock shelter with painting of red camelids. Test pits show that the shelter started to be occupied since the Formative period based on the unpainted surface of the pottery and a triangular-shaped projectile point with notch in the base; (2) Pukara 2, another rock shelter with paintings, both located at the base of a cliff; (3) Pukara 3, a group of 8 stone funerary cists on the pampa above the cliff and near Pukara 1; (4) Pukara 4, a circular stone structure, possibly used as a domestic structure (archaeological test pit yielded few stone artifacts made of basalt), located on the border of the cliff; (5) Pukara 5, another group of 3 stone funerary cists close to Pukara 3, containing scarce disturbed human remains with no offering remains; (6) Pukara 6, a group of 5 stone funerary cysts above Pukara 2; (7) Pukara 7, a small cemetery next to Pukara 1; (8) Pukara 8 stone wall corral; (9) Pukara 9 stone wall corral; (10) Pukara 10 habitational stone structures; (11) Mullutani 1, a rock shelter closed with a stone wall for corral use; (12) Millutani 2, a rock shelter with human remains, and (13) Angostura 1, a another stone walled rock shelter used as a corral.

At Pukara 6 (Figure S1B), the cysts show the same pattern of construction, characterized by stones that were vertically placed at the base, covered with more rounded stone, forming small monticules with a small opening or doorway. Although no diagnostic artifacts were found, we estimated that the cists were built during the Late Intermediate period (LIP), as they resemble a pre-Inca burial chamber described by Hyslop (1977) for the Lake Titicaca basin. During that period, local polities in the lowlands developed a regional social system recognized by a conspicuous iconography depicted in several media of the material culture (pottery, textile, rock art, basketry) (Castro et al., 2016).

Of the five tombs identified at Pukara-6 (Figure S1C), Tombs 1 and 4 were excavated. The osteological remains analyzed in this study were found in Tomb 1, and correspond to an incomplete male individual, over 40 years old at death. The osteological remains of this individual are poorly preserved and highly weathered. The osteological remains were associated with red ocher. No other cultural remains were found. The cyst is characterized by a circular shape stone structure formed by a single row of vertical slabs sticking out from the surface, no more than 60 cm. The diameter of the cyst was less than 1 m (Santoro and Standen, 1999).

##### Iroco (Bolivia): 1260-795 BP

• I01: 1260-991 calBP (1160 ± 63 BP, AA-84154)
• I02: 1175-795 calBP (1060 ± 62 BP, AA-84155)

The site of Iroco KCH11 (Figure S1D), at 3692 m above sea level, is located in the Central Altiplano, near the northern shore of Lake Uru-Uru in the Bolivian department of Oruro (Capriles, 2014). It is a 2.5 m mound that covers approximately 0.85 ha and is situated over an alluvial plain. It has a series of superimposed Wankarani Formative and Tiwanaku Period domestic occupations. The two individuals reported here are associated with slab-stone burial tombs associated with the final use of the site for funerary purposes. Excavations at the site were carried out in 2003 and 2007. The 2007 excavations focused on the central portion of the site and covered a surface area of 17 m^2^ (see Figure below). The sequence of these excavations begins with a dense and highly organic silty midden that included high densities of faunal remains, ceramics, and lithic fragments. The eastern side of the unit included a disturbed clay surface of a possible semicircular structure AMS radiocarbon dated to 974-854 calBP and surrounded by abundant faunal remains including a possible ritual offering.

The slab-stone burials were constructed cutting the sandy sediment and the midden, but the burials seem to be associated with a possible offering of well-preserved disarticulated and fragmented large camelid bones that were deposited as a result of a discrete food consumption event. The burials were oriented north-to-south, and except for two turquoise beads they did not include any grave goods. Each burial contained a single adult male individual between 35 and 45 years old with cranial modification, placed on its back and in flexed position. In addition, a third burial was identified in the northeastern corner of the excavation, consisting of a juvenile individual directly buried in a pit without slab stones. AMS radiocarbon dates from teeth of the individuals excavated from the first two burials date them to the Tiwanaku Period, between 1095-831 calBP, and it is unclear but unlikely that these individuals originated from the Titicaca area. These individuals were most likely highland llama pastoralists who also engaged in limited hunting, gathering and cultivation of chenopods and tubers (Capriles, 2017).

##### Miraflores, La Paz (Bolivia): 1250-950 BP

• MIS3: 1185-1010 calBP (1190 ± 25 BP, PSUAMS-2141)
• MIS5: 1180-985 calBP (1165 ± 25 BP, PSUAMS-2142)
• MIS6: 1250-950 BP
• MIS7: 1250-950 BP

The Miraflores site (3600 m asl), also called Putu Putu, covers approximately 30 ha and is located 60 km east of the archaeological complex of Tiwanaku, within the urban area of the city of La Paz and the middle valley of the La Paz River C.L.A., K.A.A., and E. Arratia, unpublished data. This valley was used intensively as an agricultural and residential area from as early as the Formative Period (2050-1450 BP) by people who seem to have been culturally related to both people from yungas lowlands and the Altiplano Highlands. At this time links with Tiwanaku and other Lake Titicaca communities intensified, leaving evidence in the ceramic material collected in excavations of the site.

The investigation from which the samples reported here were derived was conducted between 2015-2016 in the central area of the Miraflores site and exposed the remains of residential, ritual and funeral spaces. Remains of structures with courtyards and ritual and funeral spaces were extensively exposed, from which samples of charcoal and bone tissue were collected.

The MIS3 sample was obtained from an adult (30-40 years old) male (confirmed genetically) buried directly under the floor of a courtyard of a residential complex of Tiwanaku affiliation. The individual was found in a sitting position with legs flexed and the arms curled around the legs. His head was orientated east and on the burial a slab and a set of boulders were placed, which collapsed and covered the chest and limbs of the body. The individual was covered by a camelid fleece textile, possibly placed on the body sitting on a kind of vegetable fiber basket as suggested by imprints documented during excavations. The burial (Figure S1E) was also associated with a utilitarian undecorated ceramic vessel and a small ceramic fragment of a typical Tiwanaku keru painted with a polychrome radiated anthropomorphic face.

MIS5 corresponds to an individual buried in a cist or sillar-type tomb (Figure S1F), in a seated position, with the lower limbs bent, the head facing west and the arms folded and crossed at the level of the chest. Although no ceramic offering artifacts were found inside the tomb, radiocarbon dating and the context of the residential floors and materials associated with nearby offering pits indicates that the individual probably lived during the more consolidated Tiwanaku presence in the region. The tomb was lined with granite and ovoid quartzite rocks, which include worn ground stones, grinding stones, and other rocks that were previously used for domestic purposes. The upper cover was made up of one or two ground stones, which were subsequently removed, allowing the entry of clay sediments and rocks that covered the body of the individual and destroyed part of the skull and ribcage. The tomb was lined by a clay cover, clearly different from the ground where it was buried. In the upper part of the tomb and in the interstices of the granite rocks that formed part of the walls, an instrument made from taruca deer (*Hippocamelus antisensis*) antlers was found along with typical Tiwanaku ceramics.

Individual 6 (MIS6), corresponds to Locus 827, Feature 7 and is a secondary burial in poor condition located under a collapsed wall. Due to the high degree of deterioration, most of the bones recovered correspond to fragments of the maxilla, teeth, and long bones. Although this burial had no direct association with ceramic material, it was immersed in a matrix that included Tiwanaku type ceramics. Next to it, there was a small informal hearth, made up of a high concentration of charcoal, ash, burnt rocks, ceramics, and a charred complete maize cob C.L.A., K.A.A., and E. Arratia, unpublished data.

Individual 7 (MIS7), corresponds to Locus 22, which comprises a set of disjointed bone remains of two individuals, which were scattered in the matrix of a pit containing substantial waste. For instance, finely decorated Tiwanaku ceramic fragments were found including a couple of keru fragments (Figure S1G) decorated with the representation of the so-called “frontal-faced deity.”

##### Tiwanaku, La Paz (Bolivia): 1300-750 BP

• I0979: 1300-950 BP
• I0977: 1050-925 calBP (1056 ± 23 BP, OxA-31463)
• I0976: 1050-750 BP
• I0978: 935-795 calBP (969 ± 28 BP, OxA-31443)

Tiwanaku is a pre-Columbian archaeological site in western Bolivia. The Tiwanaku culture constitutes one of the first large-scale political entities in the Central Andes of South America and developed in the Southern basin of the Titicaca Lake. All samples were collected in the proximity of the Akapana Pyramid. The Tiwanaku site was the most important pilgrimage destination and ceremonial center of this culture, with a permanent influx of surrounding populations showing the importance of its administrative power and influence over its large territorial expansion during the Middle Horizon (1350-950 BP).

Individuals I0976 and I0977 were found in a multiple burial in the northwestern sector of the pyramid. The burial contained three human skulls (one infant, another juvenile and a third adult), the latter showed a partial cranial deformation. Disarticulated bones, vertebrae, ribs and a part of a spine (which still had partial joints) were identified, and thus this is likely to have been a secondary burial. The burial lacked grave goods. It is common to assign secondary burials like this one to a Tiwanaku affiliation.

Individual I0978 was represented as a single skull found at the western side of the pyramid, with a circular trepanation on the upper part; just to the north another human skeleton was found. The decoration of the ceramic material was assigned to the Tiwanaku V, but could be related to a late occupation of the site.

Individual I0979 is represented by the skull of a child found at the southern wall of the pyramid. The burial corresponds to a child sacrifice at the Akapana pyramid. The burial is described as a primary-type offering of just one individual. The child was found in a flexed left lateral position with an East-to-West orientation and the skull had a North-to-South orientation. In that same level of occupation there was a hind leg camelid bone, a camelid torso and coal on the same surface. Other materials included ceramic fragments with a strong density of coal dispersed and concentrated next to the offering, as well as two cores of green quartzite plus a core of greenish clay that might be related to “phasa” or Ch’akho (edible clay).

##### Monte Grande, Nasca (Peru): 965-905 BP

• I2550: 965-905 calBP (1065 ± 20 BP, PSUAMS-1905)

Monte Grande is one of the largest archeological sites in the lower part of the Rio Grande, in a small oasis near the Coast, where archaeological occupations belonging to different time periods have been recorded. The most important occupation phases are associated with the Nasca and Wari cultures. As a site located near the ocean, people at Monte Grande were engaged in exploitation of marine resources, which were exchanged as far as to the Laramate region in the Highlands. The site occupies a large alluvial terrace and the slopes of the hills that border the oasis, where there are remains of some adobe constructions, enclosures made with stone walls, and others with posts and quincha walls, living areas and cemeteries. The samples analyzed in this study come from one of these cemeteries dating to the end of the Middle Horizon.

##### Los Molinos, Palpa (Peru): 625-515 BP

• I1479: 625-525 calBP (595 ± 15 BP, PSUAMS-1605)
• I2549: 560-515 calBP (580 ± 20 BP, PSUAMS-1616)

The Los Molinos site is located in the middle section of the Rio Grande valley, near the current city of Palpa where the valleys of Palpa (Rio Grande, Palpa and Viscas) converge, forming large fields surrounded by desert plains. The famous Nasca geoglyphs are located in this region (Reindel and Isla, 2001). This area at the foot of the Andes harbors a remarkable concentration of archaeological sites.

Los Molinos is one of the main settlements in the Palpa Valley, whose most important occupation dates to the Early Nasca period (1950-1650 BP) although it was occupied until the beginning of the Middle Horizon (1300-1150 BP). The individuals reported here date to the Late Intermediate Period (950-550 BP), which means they are likely intrusive burials. The site presents a central sector with public and ceremonial architecture made with solid adobe walls, where enclosures, patios and passages arranged on terraces stand out. In areas adjacent to this part of the site there are housing spaces and cemeteries of both Nasca and the Middle Horizon. The samples studied here come from Late Nasca burials at the site.

##### Laramate Highlands (Peru): 775-540 BP

• I1396: 635-540 calBP (629 ± 19 BP, MAMS-27352)
• I1358: 775-680 calBP (875 ± 20 BP, PSUAMS-1604)
• I0042: 735-670 calBP (820 ± 24 BP, MAMS-12301)
• I1356: 640-545 calBP (640 ± 20 BP, PSUAMS-1613)

Laramate does not describe a single archaeological site but an area located in the upper part of the Palpa valleys, on the western slope of the Andes, between 2500 and 4350 m above sea level. Laramate itself includes the river basin of the same name that is a tributary of the Viscas river. The area is characterized by the presence of a series of small streams that converge in the river forming small oases of greenery surrounded by higher hills. Archaeological investigations in the area have revealed the existence of a long cultural history, starting with an occupation of hunters and gatherers from ∼10000 BP (based on archaeological evidence found in a rock-shelter of the Llamocca hill) until the Inca occupation ∼420 BP. Although much remains to be investigated, the studies developed by the Palpa-Lucanas Project have revealed occupations related to cultures such as Paracas, Nasca, Wari and Ica-Chincha, the first two and the last corresponding to cultural traditions of the Coast, while the third is related to the Highlands.

The sites in Laramate include various settlements, some quite large, with evidence of public and ceremonial activities, as well as other housing, which are mostly located on the top of the hills, in high positions and with great command of the landscape. Another type of site includes a series of rock shelters and chulpas or kuntis (funerary structures) where numerous burials have been documented. Most of the burials (which are multiple burials) date from the Middle Horizon to the Late Intermediate Period. The samples analyzed in the present study come from such Middle Horizon and Late Intermediate Period contexts. For more detailed descriptions of the long documented cultural process in the mountains refer to Isla and Reindel (2017).

##### Charangochayoc (Peru): 850-500 BP

• I2544: 850-500 BP

The Charangochayoc communal tomb, at 3584 m above sea level, consists of a structure with small rectangular rooms, with white plastered walls and rectangular windows or access points, built in a rock-shelter on the western margin of the Chicha / Soras valley. The tomb comprised 4 or 5 separate chambers and covered an area measuring 9 by 4 m. The location of this tomb is at the base of the vertical cliff type columnar basalt formation. The tomb is close to the point where a possible irrigation canal, which may have provided water to the terracing surrounding the lower-lying Middle Horizon site of Yako, dropped into the valley (Meddens and Cook, 2001). The remains of approximately 200 individuals, including fragments of mummy bundles, were found here, with skull deformation being a common feature. W.H. Isbell defined this type of tomb using the term ‘Open Sepulchre’ (Isbell, 1997). The associated ceramic complex at Charangochayoc includes Viñaque, Huamanga and Black Decorated style components as well as a local Chicha style material. The latter becomes dominant during the Middle Horizon period 3-4 and continued into the Late Intermediate Period.

##### Mesayocpata (Peru): 550-390 BP

• I2545: 550-390 BP

Mesayocpata, at 4349 m above sea level, has a single tier ushnu platform measuring 18.1 × 9.8 m with a wall width of ∼0.65 m. The wall height above the level of the turf is ∼0.74 m. The wall construction is of cut stone polygonal ashlar, and a row of corbels projects from the top of the wall. The stonework is characteristic of the Late Horizon Inca tradition.

Southwest of the platform were found the remains of a badly damaged circular chullpa constructed of modified field stone. The structure has a diameter of 2.6 m across and the walls are 55-60 cm wide. The remains of an entrance are present on the southeast side. Some badly eroded human bone was found inside comprising a single right femur with the proximal epiphysis fused (c. 36 × 36 mm) and the distal joint missing. Fragmentary skull elements were also present.

South of the platform a further chullpa is located. It has an exterior diameter of 2.6 m with a wall width of 60 cm. An entrance is present on the southeast side. This is 75 cm high and 44 cm wide. The dome shaped roof is largely in place. Three Saywas are located along the bedrock outcrop rising up and overlooking the platform from this position. South of the platform a number of Huanca stones are found. Segments of three or possibly four roads of potential Late Horizon date pass by to the east.

##### Ullujaya, lower Ica Valley (Peru): 1480-920 BP

• I2560: 1045-920 calBP (1088 ± 24 BP, OxA-26975)
• I2557: 1480-1320 calBP (1558 ± 25 BP, OxA-26973)
• I2558: 1365-1275 calBP (1455 ± 32 BP, OxA-26974)

The archeological record of the Ica Valley reflects the rich cultural efflorescence and subsequent cultural collapse on the South Coast of Peru, a region defined by often shared cultural heritage across several adjacent river valleys, including the Nasca drainage to the south. It is also the location of the Monte Grande, Nasca and Los Molinos (Palpa) sites described above. The Ullujaya Basin is one of a sequence of riverine oases that define the lower courses of the Ica River, the western edges of which are lined with extensive cemeteries dating to various time periods from the Early Horizon through to the Inca Late Horizon. These have been severely looted and yet their human remains have yielded important information on changing diet over almost two millennia (Cadwallader, 2013). Three individuals from Ullujaya in this study derive from two such contexts: individual I2560 from Cemetery 398, directly dated to the Middle Horizon; and individuals I2557 and I2558 from Cemetery 734 directly dated to the Late Nasca Period (Samples 91, 9 and 14, respectively, in Cadwallader et al. [2015]: 269, and see Cadwallader et al. [2018] for full details on the locations and distinctive associated funerary architecture and material culture of these burials).

##### Campanayuq (Peru): 1150-850 BP

• I2236: 1150-850 BP
• I2543: 965-920 calBP (1085 ± 20 BP, PSUAMS-1620)
• I2563: 1150-850 BP

Campanayuq Rumi is at an elevation of 3600 m above sea level in Vilcashuaman in the Ayacucho Region. The civic-ceremonial center dating to the Initial and Early Intermediate Period was first systematically excavated in the late 2000s (Matsumoto et al., 2013), and exhibits architecture that closely resembles the contemporary site of Chavin de Huantar, ∼600 km to the north (Nesbitt et al., 2019). The skeletons reported here date to the Late Intermediate Period long after the main occupation phase of the site. The individuals were found close to the original site. The radiocarbon dates reported here confirm that they are intrusive burials not directly associated with the site.

##### San Sebastian, Cusco (Peru): 665-465 BP

• I1743: 520-465 calBP (425 ± 20 BP, PSUAMS-1611)
• I1744: 665-555 calBP (640 ± 20 BP, PSUAMS-1612)
• I1400: 655-550 calBP (615 ± 20 BP, PSUAMS-1610)

The San Sebastian site was an Inca site in Cusco, which was destroyed by the urban expansion of the city. The skeletons studied here were part of the Yale Bingham collection and have been repatriated to Cusco, Peru.

##### Torontoy, Cusco (Peru): 512-417 BP

• TOY-1-1: 512-417 BP
• TOY-4-5: 512-417 BP
• TOY-7A-1: 512-417 BP

The Torontoy site in Peru is located on the right bank of the Urubamba drainage approximately 83 km northwest of the city of Cusco and within the Machu Picchu state park. It is characterized by classic Inca stone architecture including trapezoidal doors and niches, tightly fitted masonry, patios, multi-room compounds, and a stone bath chamber. This allowed cultural dating of these individuals to the Inca period (512-417 BP). It has been investigated by archaeologists of the Peruvian Ministry of Culture. The burials were found during the excavation of the structures.

##### Huaca Pucllana, Lima (Peru): 1850-480 BP

• I0969: 885-545 calBP (955 ± 65 BP, OxA-31423)
• I0972: 1050-480 BP
• I0964: 635-480 calBP (745 ± 23 BP, OxA-31424)
• I0965: 650-495 calBP (773 ± 24 BP, OxA-31425)
• I0967: 1050-480 BP
• I0966: 1050-480 BP
• I0044: 720-535 calBP (866 ± 28 BP, OxA-31119)
• I0971: 1450-1050 BP
• I0968: 965-755 calBP (1156 ± 22 BP, OxA-31422)
• I0974: 1750-1250 BP
• I0975: 1315-1090 (1493 ± 29 BP, OxA-31120)
• I0045: 1850-1300 BP

Huaca Pucllana is a pyramid complex that is now situated in the Miraflores section of Lima. Its main pyramid is built of small adobe bricks. It is considered one of the major surviving sites of the Lima culture, an Early Intermediate Period culture dating to between 1950-1250 BP. There is much evidence of ritual activity on the patios, terraces and flat-topped summit of the pyramid complex during this time, including feasting and sacrificial offerings. Following its abandonment, the complex was used as a burial ground for Middle Horizon populations that were under the influence of the Wari polity. As the radiocarbon results show, the burials sampled included individuals from the Lima culture and Middle Horizon components, as well as later groups like the Late Intermediate Period Ychsma. For further details refer to Valverde et al. (2016).

##### Chinchawas (Peru): 1200-550 BP

• I2252: 1200-850 BP
• I2253: 1200-850 BP
• I2250: 850-550 BP
• I2251: 850-550 BP
• I2264: 850-550 BP

Chinchawas was a small prehispanic village and ceremonial center in the Cordillera Negra (Distrito de Pira, Provincia de Huaraz, Ancash). It was settled originally by ∼1650 BP and its occupation extended into early colonial times. Its most intensive use (1450-1050 BP) was by groups of the Recuay tradition, based on evidence of pottery, architecture, stone sculpture, and local economic and funerary practices.

Located at 3850 masl, the village prospered through cultivation of high altitude crops, herding and trade (Lau, 2010). The herding economy was especially important, as seen through abundant remains of camelids, spindle whorls for spinning fiber, weaving tools, and small camelid effigies dedicated to animal fertility. Long-distance trade of obsidian, exotic pottery and metalwork, especially during the Middle Horizon, may also indicate exchange, perhaps reliant on llama caravans. Interregional contacts included stylistic interaction with Cajamarca, Lima, Wari, Late Moche and other prestige styles of the Central Andes (Lau, 2005). Chinchawas’s favorable location along a major Coast–Highland trade route (Casma-Huaraz) was a main reason for the establishment and prosperity of the ancient community.

The range of exchange contacts belies the small size of Chinchawas, which is comprised of a principal sector (about 4 hectares) of stone house constructions and ceremonial constructions (small walled enclosures and a tall circular building housing a rock outcrop). An adjacent sector consists of a cemetery, located to the east and southeast of the village. The teeth samples derived primarily from interments in above-ground tombs (chullpas). These multi-interment structures housed mummified corpses, plausibly of related people. Human bones sometimes clustered into groups, but most were found disarticulated. Pottery and other artifacts suggest that chullpas were first used in the Middle Horizon (∼1250 BP), displacing the earlier Recuay practice favoring subterranean tombs and continuing well into the Late Intermediate Period (950-550 BP). The individuals represented by the teeth samples were of Middle Horizon and Late Intermediate Period peoples. A range of ceramic styles suggest strong stylistic ties with groups from different areas, but especially with the Central and North Coasts. One of the notable aspects of Chinchawas is the strong evidence linking stone sculptural production with ceremonial practices associated with burial and veneration activities.

##### El Brujo Archaeological Complex (Peru): 1750-560 BP

• I2241: 1200-650 BP
• II2242: 765-560 calBP (965 ± 20 BP, PSUAMS-1606)
• I2243: 1200-650 BP
• I2244: 1200-650 BP
• I2237: 1425-1245 (1650 ± 20 BP, PSUAMS-1607)
• I2238: 1750-1350 BP
• I2262: 1750-1350 BP
• I2263: 1240-940 calBP (1390 ± 15 BP, UCIAMS-186351)
• I0324: 1240-935 calBP (1388 ± 18 BP, MAMS-25006)

The El Brujo Archaeological Complex is situated at the Peruvian northern coast in the Chicama Valley, ∼40 km north of Trujillo. The complex includes several sites of which the most prominent are Huaca Prieta, with the oldest known evidence of human presence at the Peruvian coast (∼14,000 BP) (Dillehay et al., 2012), and the Huaca Cao Viejo, a stepped truncated pyramid dating into the Early Intermediate Period, built by the Moche (1850-1250 BP), and containing the remains of the Senora de Cao (Cesareo et al., 2018, Quilter, 2013). While most of the archaeological structures at the complex are associated with the Moche occupation, there are also a number of sites associated with the subsequent Lambayeque and Chimu archaeological cultures. The samples studied here derive from Moche and Lambayeque associated burials throughout the El Brujo complex. None of the individuals were buried in the huacas (pyramids), indicating that they are not of elevated social status.

##### Laguna Chica (Argentina): 1700-1565 calBP

• I8351: 1700-1565 calBP (1750 ± 15 BP, UCIAMS-185301)

The Laguna Chica archaeological site is located on the margins of a seasonal lake in the southeast of the Hinojo-Las Tunas Shallow Lake System. The study area belongs to the Central Pampas Dunefields unit of the aeolian system of central Argentina. Six burials were identified: three in Sector A (Burial N 1, Burial N 2, Burial N 6) located in the southern part of the shallow lake and three in Sector B (Burial N 3, Burial N 4, Burial N 5) in the west area (Messineo et al., 2019). The inhumations were dated to the Middle and Late Holocene.

Burial N_2 contained two articulated individuals in a dorsal position with the lower limbs flexed. Individual N_1 (LCH.E2-I1.2, sample SC50-L762 in the ancient DNA study of Posth et al. [2018]) is an adult female and was dated to 6780-6650 calBP. Individual N_ 2 (LCH.E2-I2.1, sample SC50-L761 in Posth et al. [2018]) is an adult male and was dated to 6960-6790 cal BP. Burial N 4 (LCH.E4.4; SC50_L764) is an infant of undetermined sex. This individual was dated to 1700-1565 calBP.

Although no stratigraphic excavations have been performed in the site, besides the burial, abundant lithic material has been found on the surface along the beach of the lake. These lithic materials are characterized by a predominance of orthoquartzite, followed by other lithic raw materials in low frequencies such as chert, granites, basalt, siliceous chert, and silex, among others. There is a high diversity of tools such as side-scrapers, end-scrapers, knives, multipurpose tools, triangular projectile points, and others. In addition, the excavation recovered exhausted orthoquartzite cores. Most lithic raw material came from the Tandilia hill range system (250-350 km to the southeast), but a small quantity of rock came from the Ventania hill range system (170 to the km south), the Tehuelche Mantle (300 km to the southwest), and the Dry Pampas (480 km to the west). The preliminary analysis of the material indicates that the site was occupied during Middle and Late Holocene times. It might represent a succession of residential camps in the border of the pond by hunter-gatherers focused in the exploitation of guanaco (*Lama guanicoe*).

### Methods Details

#### Direct AMS ^14^C bone dates:

We report 39 new direct AMS ^14^C bone dates from 5 radiocarbon laboratories (Arizona [AA] – 2; Mannheim [MAMS] – 5; Oxford Radiocarbon Accelerator Unit [ORAU] – 11; Pennsylvania State University [PSUAMS] – 17; UC Irvine [UCIAMS] - 4) (Table S1). Bone preparation and quality control methods for most of these samples are described elsewhere. Methods for each lab are described in the following: Arizona: (Capriles, 2014); Mannheim: (Posth et al., 2018); Oxford: (Cadwallader et al., 2015, Valverde et al., 2016); PSUAMS: (Olalde et al., 2019); UCIAMS: (Beverly et al., 2010).

#### Calibration of radiocarbon dates:

All calibrated ^14^C ages were calculated using OxCal version 4.3 (Ramsey and Lee, 2013). Northern or southern hemisphere calibration curves used were based generally on the position of the summer Intertropical Convergence Zone (ITCZ) rather than the geographic hemisphere following recent archaeological studies in this region (Marsh et al., 2018). The IntCal13 northern hemisphere curve (Reimer et al., 2013) was used for samples within the Amazon Basin and Altiplano, while the remainder were calibrated using the SHCal13 curve (Hogg et al., 2013). Dates from coastal sites were calibrated using a mixture of SHCal13 with the Marine13 curve (Reimer et al., 2013) based on an estimate of a 40% marine dietary component. For each site, ΔR values were calculated based on the most proximate sample locations in the 14CHRONO Marine Reservoir Database (Reimer and Reimer, 2001) (see Table S1 for details). We recognize that there might still exist potential uncorrected biases due to the uncertainty in past carbon 14 variation in this region. However, in studies by others (Marsh et al., 2018) and our own tests with different calibrations, maximum differences between different calibration choices amount to four decades at 3400BP or a maximum of a couple of decades during the brief Inca Late Horizon. All details and error ranges for the dates and calibrations are found in Table S1.

#### Grouping of Individuals:

To define genetic group labels we generally used the following nomenclature: “*Country_SiteName_AgeBP*” (Eisenmann et al., 2018). “*AgeBP*” of a genetic group comprised of more than one individual is calculated by averaging the mean calibrated date in years before present (BP) of the directly dated samples that provided nuclear DNA data. For samples that were not directly dated we considered the averaged value of the corresponding genetic group.

#### Ancient DNA Laboratory Work:

All samples in this study were processed in the dedicated clean rooms at UCSC Paleogenomics in Santa Cruz (USA), Harvard Medical School in Boston (USA), or the Australian Centre for Ancient DNA in Adelaide in Australia (ACAD), following strict procedures to minimize contamination (Llamas et al., 2017). In all three labs, DNA was extracted from bone or tooth powder using a method that is optimized to retain small DNA fragments (Dabney et al., 2013, Korlević et al., 2015). Double-stranded sequencing libraries were prepared for most samples using previously established protocols (UCSC & Harvard [Rohland et al., 2015]; ACAD [Llamas et al., 2016]). The sequencing libraries for Iroco, Miraflores and Torontoy were built using a single-stranded library preparation method described by Troll et al. (2019). All samples were treated with uracil-DNA glycosylase (UDG) to greatly reduce the presence of errors characteristic of ancient DNA at all sites except for the terminal nucleotides (Rohland et al., 2015), or including at the terminal nucleotides (UDGplus) (Briggs et al., 2010).

We enriched the libraries both for sequences overlapping mitochondrial DNA (Maricic et al., 2010), and for sequences overlapping about 1.24 million nuclear targets after two rounds of enrichment (Fu et al., 2015, Haak et al., 2015, Mathieson et al., 2015). We sequenced the enriched products on an Illumina NextSeq500 using v.2 150 cycle kits for 2 × 76 cycles and 2 × 7 cycles, and sequenced up to the point so that the expected number of new SNPs covered per 100 additional read pairs sequenced was approximately less than 1. Enrichment was performed either at Harvard Medical School (USA), or the Max Plank Institute for Science of Human History in Jena (Germany).

To analyze the data computationally, we merged paired reads that overlapped by at least 15 nucleotides using SeqPrep (https://github.com/jstjohn/SeqPrep) taking the highest quality base to represent each nucleotide, and then mapped the sequences to the human genome reference sequence (GRCh37 from the 1000 Genomes project) using the *samse* command of the Burrows-Wheeler Aligner (*BWA*) (version 0.6.1) (Li and Durbin, 2010). We trimmed two nucleotides from the end of each sequence, and then randomly selected a single sequence at each site covered by at least one sequence in each individual to represent their genotype at that position (“pseudo-haploid” genotyping).

We assessed evidence for ancient DNA authenticity by measuring the rate of damage in the first nucleotide (flagging individuals as potentially contaminated if they had a less than 3% cytosine-to-thymine substitution rate in the first nucleotide for a UDG-treated library and less than 10% substitution rate for a non-UDG-treated library). To determine kinship we computed pairwise mismatch rates between the different individuals following the same approached used in Kennett et al. (2017).

### Quantification and Statistical Analysis

#### Contamination estimation in mitochondrial DNA, the X chromosome, and the autosomes:

We estimated mtDNA contamination using *contamMix* version 1.0-12 (Fu et al., 2013), which creates a Bayesian estimate of a consensus sequence composed of the true ancient DNA, error and contamination, which could come from any of a set of current human full-length mitochondrial genomes that span all plausible contaminating sequences. The software was ran with down-sampling to 50x for samples above that coverage,–trimBases X (2 bases for UDG-half samples and 10 bases for UDG-minus samples), 8 threads, 4 chains, and 2 copies, taking the first one that finished. For males we estimated X chromosome contamination with ANGSD (Korneliussen et al., 2014), which is based on the rate of heterozygosity observed on the X chromosome. We used the parameters minimum base quality = 20, minimum mapping quality = 30, bases to clip for damage = 2, and set all other parameters to the default. Lastly, we measured contamination in the autosomes using *ContamLD*, a tool based on breakdown of linkage disequilibrium that works for both males and females (Nakatsuka et al., 2020). We report but do not include in our main analyses samples with evidence of contamination greater than 5% by any of the contamination estimation methods (samples I1400, I01, and MIS6 were excluded). All contamination estimates are reported in Table S1.

#### Present-day human data:

We used present-day human data from the Simons Genome Diversity Project (Mallick et al., 2016), which included 26 Native American individuals from 13 groups with high coverage full genome sequencing. We also included data from 224 Native American individuals from 34 different populations genotyped on the Affymetrix Human Origins array (Barbieri et al., 2019, Lazaridis et al., 2014, Skoglund et al., 2015) as well as 493 Native American individuals genotyped on Illumina arrays either unmasked or masked to remove segments of possible European and African ancestry (Reich et al., 2012).

#### Y chromosome and mitochondrial DNA analyses:

For Y chromosome haplogroup calling, we used the original BAM files and performed an independent processing procedure. We filtered out reads with mapping quality < 30 and bases with base quality < 30, and for UDGhalf treated libraries we trimmed the first and last 2 bp of each sequence to remove potential damage induced substitutions. We determined the most derived mutation for each sample using the tree of the International Society of Genetic Genealogy (ISOGG) version 11.110 (accessed 21 April 2016) and confirmed the presence of upstream mutations consistent with the assigned Y chromosome haplogroup, manually checking each of the haplogroups.

To identify the mitochondrial haplotypes of the individuals, we manually analyzed each variant as described in (Llamas et al., 2016) rather than relying on automated procedures. All mitochondrial reads mapped to the rCRS or RSRS using BWA were visualized in Geneious v7.1.3 (Biomatters; available from https://www.geneious.com/) for each sample. Initially, SNPs were called in Geneious for all polymorphisms with minimum coverage 5 and a minimum variant frequency 0.8. The assembly and the resulting list of SNPs were verified manually and compared to SNPs reported at phylotree.org (mtDNA tree Build 17 [18 Feb 2016]) (van Oven, 2015). Following recommendations in van Oven and Kayser 2009 (van Oven and Kayser, 2009), we excluded common indels and mutation hotspots at nucleotide positions 309.1C(C), 315.1C,AC indels at 515–522, 16182C, 16183C, 16193.1C(C), and C16519T. We embedded the consensus mitochondrial genomes in the existing mitochondrial tree (mtDNA tree Build 17 [18 Feb 2016]) using the online tool HaploGrep2 (Weissensteiner et al., 2016) to determine the haplotypes.

We generated a multiple genome alignment of 45 newly reported mtDNA sequences (excluding sample IO1 because of low coverage) together with 91 previously published ancient mtDNAs from western South America (Fehren-Schmitz et al., 2015, Llamas et al., 2016, Posth et al., 2018) and 196 modern-day sequences (Llamas et al., 2016) using MUSCLE (parameter: -maxiters 2) (Edgar, 2004). The complete aligment cosists of 333 mtDNA sequences belonging to haplogroups A, B, C and D, plus an haplogroup L3 sequence as outgroup. The program MEGA6 (Tamura et al., 2013) was used to construct a Maximum Parsimony tree with 99% partial deletion (16543 positions) and 500 bootstrap iterations that was visualized with FigTree (http://tree.bio.ed.ac.uk/software/) (Figure S2). This tree recapitulates the star-like phylogeny of the founding Southern Native American mtDNA haplogroups reported previously (Tamm et al., 2007).

#### ADMIXTURE clustering analysis:

Using PLINK2 (Chang et al., 2015), we first pruned our dataset using the–geno 0.7 option to ensure that we only performed our analysis on sites that had at least 70% of samples with a called genotype. We then ran ADMIXTURE (Alexander et al., 2009) with 100 replicates for each K value, reporting the replicate with the highest likelihood. We show results for K = 2 to 18 in Figure S3. Replications and automated filtering were performed using the UCSC-PL wrapper script adpipe.py (https://github.com/mjobin/UPA/blob/master/adpipe.py).

#### Principal Components Analysis:

We performed principal components analysis (PCA) using the *smartpca* version 16680 in EIGENSOFT (Patterson et al., 2006). We used the default parameters and the lsqproject: YES, and newshrink: YES options and performed PCA on the Human Origins dataset of present-day un-admixed Andean individuals (Barbieri et al., 2019). We projected the ancient individuals onto the principal components determined from the present-day individuals. When plotting the principal components we reversed the eigenvector 1 values so that the strong correspondence to the geography of Peru would be more apparent.

#### Symmetry statistics and admixture tests (f-statistics):

We used the *qp3pop* and *qpDstat* packages in ADMIXTOOLS (Patterson et al., 2012) to compute *f*_*3*_-statistics and *f*_*4*_-statistics (using the f4Mode: YES parameter in *qpDstat*) with standard errors computed with a weighted block jackknife over 5-Mb blocks. We used the inbreed: YES parameter to compute *f*_*3*_-statistics to account for our random allele choice at each position (due to having too little data to determine the full diploid genotype). We computed “outgroup *f*_*3*_”-statistics of the form *f*_*3*_*(Mbuti; Pop1, Pop2)*, which measure the shared genetic drift between population 1 and population 2. We created a matrix of the outgroup-*f*_*3*_ values between all pairs of populations. We converted these values to distances by subtracting the values from 1 and generating a multi-dimensional scaling (MDS) plot with a custom R script. We converted the original values to distances by taking the inverse of the values and generating a neighbor joining tree using PHYLIP version 3.696’s (Felsenstein, 1989) neighbor function and setting *USA-MT_Anzick1_12800BP* as the outgroup (default settings were used for the rest of the analysis). We displayed the tree using Itol and set all of the tree lengths to “ignore” (Letunic and Bork, 2011). In some of our analyses we plot the *f*-statistics on a heatmap using R (https://github.com/pontussk/point_heatmap/blob/master/heatmap_Pontus_colors.R).

#### Grouping ancient samples into analysis clusters:

The ancient individuals were first grouped by archaeological site and time period based on archaeological designations (EIP = Early Intermediate Period, MH = Middle Horizon, LIP = Late Intermediate Period, LH = Late Holocene) (Figure 1B). We ran *qpWave* and computed statistics of the form *f*_*4*_*(Mbuti, Test, Individual 1, Individual 2)* iterating over all possible pairs of individuals in each group. For the *f*_*4*_-statistics we looked for asymmetries with any external group as *Test*. For *qpWave* analyses, we tested for evidence of two sources of ancestry relative to outgroups, which were one randomly chosen group from each geographic region outside of the region where the group was from (Table S5). We did not find evidence for more than one source of ancestry in any of the pairs except in Cusco unless a group from that geographic region (Figure 1B) was added to the outgroup set. We then performed the same procedure for pairs of groups in the same geographic region and found that we could not detect significant heterogeneity within a geographic region except in Cusco and the Titicaca Basin. Beyond the regions in Figure 1B, we could not cluster the groups further, because the *qpWave* analyses showed evidence for heterogeneity when comparing groups from different regions. For Cusco (Torontoy and SanSebastian), we kept the individuals as separate except where indicated in the text.

#### qpWave analyses:

To determine the minimum number of sources of ancestry contributing to Central Andes groups, we used *qpWave* (Reich et al., 2012), which assesses whether the set of *f*_*4*_-statistics of the form *f*_*4*_*(A = South American 1, B = South American 2; X = outgroup 1, Y = outgroup 2)*, which is proportional to the product of allele frequencies summed over all SNPs *(p*_*A*_*-p*_*B*_*)(p*_*X*_*-p*_*Y*_*)*, forms a matrix that is consistent with different ranks (rank 0 would mean consistency with a single stream of ancestry relative to the outgroups; rank 1 would mean 2 streams of ancestry, etc.). The significance of the statistic is assessed using a Hotelling T^2^ test that corrects for the correlation structure of *f*_*4*_-statistics (and thus multiple hypothesis testing). For all *qpWave* analyses, we used the default settings except for the change that we set allsnps: YES. For analyses to determine the number of waves of ancestry from North America, we used ancient California individuals from Scheib et al. (2018) (*USA_MainlandChumash_1400BP and USA_SanClemente-SantaCatalina_800BP*), *Russia_MA1_24000BP (MA1), USA-MT_Anzick1_12800BP, Papuan, Karelia Hunter Gatherer*, and modern Mexican groups (*Zapotec, Mixtec,* and *Mayan*) as outgroups.

#### Admixture Graph analyses:

We used *qpGraph* (Reich et al., 2009) in ADMIXTOOLS to model the relationships between the different groups. For all analyses we removed transition SNPs at CpG sites and used default settings with outpop: Mbuti.DG and useallsnps: YES. We removed individuals I0044 and I0042 from these analyses due to their different processing in the laboratory (shotgun sequencing), which created artificial evidence of shared ancestry with similarly processed shotgun-sequenced outgroup populations. We used a previously published skeleton graph for Native Americans (Lipson and Reich, 2017, Skoglund et al., 2015) and successively added in additional populations in all combinations, allowing up to one admixture from the existing groups. We took the graph with the lowest maximum Z-score and then repeated the process, adding another population until all populations of interest were added. For the main graph (Figure 4) we used the 1240K SNP set and first started with the two oldest individuals (*Peru_Lauricocha_8600BP* and *Peru_Cuncaicha_9000BP*) and then added on the individuals *Peru_Lauricocha_5800BP*, *Peru_Cuncaicha_4200BP*, and *Peru_LaGalgada_4100BP*. We then added the groups in order: *NorthPeruHighlands*, *SouthPeruHighlands*, *SouthPeruCoast*, *CentralPeruCoast*, *NorthPeruCoast*, *Bolivia_Tiwanaku_1000BP*, *NorthChile*. For the local graph (Figure S6A) to test the interactions between *NorthPeruHighlands* and *SouthPeruHighlands,* we first started with the individuals *Peru_Lauricocha_5800BP* and *Peru_Cuncaicha_4200BP*. We then added in *Peru_LaGalgada_4100BP*, then *NorthPeruHighlands* and *SouthPeruHighlands* in either order.

For the graph co-analyzing the Amazonians (Figure S6B), we used the Human Origins SNP set and first started with the structure from all individuals over 4,000 years old. We then added on *Peru_SanMartin_modern*, then *SouthPeruCoast*, and *NorthPeruCoast*. For the Argentina graph (Figure S6C) we began with *Argentina_LagunaChica_6800BP* and *NorthPeruHighlands*. We then added on *Bolivia_Tiwanaku_1000BP* and *NorthChile*, so that we had a mix of groups with differential affinity to *Argentina_LagunaChica_1600BP*. Lastly, we added in *Argentina_LagunaChica_1600BP*.

#### Formal modeling of admixture history:

We used *qpAdm* (Haak et al., 2015) in the ADMIXTOOLS package to estimate the proportions of ancestry in a *Test* population deriving from a mixture of *N* ‘reference’ populations by taking advantage of the fact that they have shared genetic drift with a set of ‘Outgroup’ populations. We set the details: YES parameter, which reports a normally distributed Z-score for the fit (estimated with a block jackknife).

To model the genetic admixture between people related to those of the Amazon and Northwest Peru, we first modeled each of the Amazonian groups as a mixture of groups related to *Peru_LaGalgada_4100BP* and *Brazil_Karitiana_modern* with the following outgroups: *Argentina_ArroyoSeco2_7700BP*, *USA-MT_Anzick1_12800BP*, *Peru_Cuncaicha_4200BP*, *Chile_Conchali_700BP*, *Mexico_Mixe_modern*, *USA-CA_SanNicolas_4000BP*, *Chile_CaletaHuelen_1100BP*, and *Brazil_Moraes_5800BP*.

We then modeled each of the Andean groups as a mixture of Peru_LaGalgada_4100BP and Peru_SanMartin_modern with the following outgroups: Argentina_ArroyoSeco2_7700BP, USA-MT_Anzick1_12800BP, Peru_Cuncaicha_4200BP, Chile_Conchali_700BP, Mexico_Mixe_modern, USA-CA_SanNicolas_4000BP, Chile_CaletaHuelen_1100BP, Brazil_Moraes_5800BP, Bahamas_Taino_1000BP.

To study the admixture between groups related to those in the Argentine Pampas and the Andes, we modeled *Argentina_LagunaChica_1600BP* as a mix of *Argentina_LagunaChica_8600BP* and, in series, one of the following groups (*Peru_Laramate_900BP*, *Chile_CaletaHuelen_1100BP*, *SouthPeruHighlands*, *NorthChile*, or *CentralPeruCoast*) with the following outgroups (*Peru_Lauricocha_5800BP*, *USA-MT_Anzick1_12800BP*, *Mexico_Mixe_modern*, *USA-CA_SanNicolas_4000BP*, *Brazil_LapaDoSanto_9600BP*, *Bahamas_Taino_1000BP*, and *Peru_LaGalgada_4100BP*).

To study the ancestries of Aymara and Quechua, we modeled *Peru_AymaraIllumina* and *Peru_QuechuaIllumina* (Reich et al., 2012) as a mixture of *Bolivia_MiraFlores_1100BP* and *CentralPeruCoast* with the following outgroups (*Argentina_ArroyoSeco2_7700BP*, *USA-MT_Anzick1_12800BP*, *Peru_Cuncaicha_4200BP*, *Chile_Conchali_700BP*, *Mexico_Mixe_modern*, *USA-CA_SanNicolas_4000BP*, *Brazil_Moraes_5800BP*, *Bahamas_Taino_1000BP, Peru_Lauricocha_5800BP*).

#### DATES (Distribution of Ancestry Tracts of Evolutionary Signals):

The *DATES* software (Narasimhan et al., 2019) measures admixture dates in DNA samples by modeling the decrease in allele covariance over genetic distance in a group relative to the allele frequencies of the two source groups. This software does not require diploid information or high coverage data and can thus work well with ancient DNA samples (unlike ALDER) (Loh et al., 2013), which measures admixture linkage disequilibrium directly and thus requires diploid information or multiple individuals, which is the equivalent). We used the default settings with jackknife: YES and used the software to analyze all potential admixtures we report in this study assuming 28.5 years per generation.

### Data and Code Availability

All sequencing data are available from the European Nucleotide Archive, accession number: PRJEB37446. Genotype data obtained by random sampling of sequences at approximately 1.24 million analyzed positions are available at the Reich lab website: https://reich.hms.harvard.edu/datasets.

## Supplemental Information

Table S1 Details of All Individuals in This Study, Related to Figure 1

Table S2: Comparisons of Highlands vs. Coast and North vs. the South, Related to Figure 4 Related to Figure 4 (A) *f*_*4*_-statistics comparing Peru Highlands groups to Peru Coast groups and (B) North Peru Highlands groups to South Peru Highlands groups.

Table S3: Comparisons within the Andes over Time and Analyses of the Argentina Inca Individual, Related to Figure 5 Related to Figure 5 (A) *f*_*4*_-statistics comparing pre-2000BP groups with post-2000BP groups, showing migrations between these time periods and (B) comparing post-2000BP groups with each other, showing genetic continuity in each region, as well as (C) *f*_*4*_-statistics, *qpWave*, and *qpAdm* analyses showing the Argentina Inca individual is of North Peruvian Coast ancestry.

Table S4 Key Statistics, Related to STAR Methods Z-scores for *f*_*4*_*(Mbuti, Row; Column_2, Column_1)*, with each row specifying one population and each column two); for example, row 1, column 2 shows *f*_*4*_*(Mbuti, WestAmazon_SanMartin_Modern; Peru_NorthHighlands_Lauricocha_5800BP, Peru_NorthCoast_1025-700BP)*. Red indicates statistics Z > 3 showing attraction of Row to Column_1. The Column_2 groups were chosen because they are geographically close to the Column_1 groups, and highlight the signals mentioned in the text. *Cusco_Torontoy_A* is the grouping of the TOY-1-1 and TOY-4-5, and *Cusco_Torontoy_B* is TOY-7A-1.

Table S5 Results of *qpWave* Analyses of Genetic Heterogeneity within Each Group, Related to Figures 1 and 2

Table S6 *f*_*4*_-statistics and *qpAdm* Analyses Comparing Ancient Individuals to Modern Individuals, Showing Genetic Continuity to the Present and Genetic Differences between Quechua and Aymara Speakers, Related to Figures 2, 3, and S6

Table S7: Statistics about the Titicaca Basin, Cusco, the Northwest Amazon, and Argentina, Related to STAR Methods Related to STAR Methods (A) *f*_*4*_-statistics, *qpWave*, and *qpAdm* analyses comparing Titicaca Basin groups with each other, showing genetic heterogeneity, and (B) modern Cusco individuals to ancient Cusco individuals, showing heterogeneity of the ancient Cusco individuals and genetic change in this region over time. (C) *f*_*4*_-statistics and results of *qpAdm* analyses of the interactions of the Central Andes with Northwest Amazon and (D) the Argentina Pampas.

Table S8 Results of *qpWave* and *DATES* Analyses of California Channel Island-Related Ancestry in the Central Andes over Time, Related to Figure S8 and STAR Methods

Data S1 English and Spanish Translation of the Summary and Key Findings Sections, Related to STAR Methods

Video Abstract
