# Supplementary material for: A Paleogenomic Reconstruction of the Deep Population History of the Andes
Source: Cell. 2020 May 28;181(5):1131–1145.e21. doi: 10.1016/j.cell.2020.04.015 (PMC7304944; doi:10.1016/j.cell.2020.04.015)
Supplement: Table S4 — Key Statistics, Related to STAR Methods Z-scores for f4(Mbuti, Row; Column_2, Column_1), with each row specifying one population and each column two); for example, row 1, column 2 shows f4(Mbuti, WestAmazon_SanMartin_Modern; Peru_NorthHighlands_Lauricocha_5800BP, Peru_NorthCoast_1025-700BP). Red indicates statistics Z > 3 showing attraction of Row to Column_1. The Column_2 groups were chosen because they are geographically close to the Column_1 groups, and highlight the signals mentioned in the text. Cusco_Torontoy_A is the grouping of the TOY-1-1 and TOY-4-5, and Cusco_Torontoy_B is TOY-7A-1. [file mmc4.pdf]

	WestAmazon_SanMartin_Modern	NorthPeruCoast_1750-560BP vs. Lauricocha_5800BP	NorthPeruHighlands_1200-550BP vs. Lauricocha_5800BP	NorthPeruHighlands_LaGalgada_4100BP vs. Lauricocha_5800BP	NorthPeruHighlands_Lauricocha_8600BP vs. Lauricocha_5800BP	NorthPeruHighlands_Lauricocha_5800BP	NorthPeruHighlands_Lauricocha_3500BP vs. Lauricocha_5800BP	CentralPeruCoast_1850-480BP vs. Lauricocha_5800BP	SouthPeruCoast_1480-515BP vs. Cuncaicha_4200BP	SouthPeruHighlands_1150-390BP vs. Cuncaicha_4200BP	Cuncaicha_9000BP vs. Cuncaicha_4200BP	Cuncaicha_4200BP	Cuncaicha_3300BP vs. Cuncaicha_4200BP	Cusco_SanSebastian_610-490BP	Cusco_Torontoy_A_450BP vs. Cusco_SanSebastian_610-490BP	Cusco_Torontoy_B_450BP vs. Cusco_SanSebastian_610-490BP	TiticacaBasin_Tiwanaku_1000BP vs. MiraFlores_11000BP	TiticacaBasin_Chile_Pukara_700BP vs. MiraFlores_1100BP	TiticacaBasin_RioUncallane_1700BP vs. MiraFlores_1100BP	TiticacaBasin_MiraFlores_1100BP	TiticacaBasin_Iroco_1050BP vs. MiraFlores_11100BP	NorthChile_1350-570BP vs. MiraFlores_1100BP	Argentina_LagunaChica_1600BP vs. Argentina_LagunaChica_8600BP	Argentina_LagunaChica_8600BP
**WestAmazon_SanMartin_Modern**	-	3.3	3.1	1.6	-1.3	-	0.8	2.7	-0.9	0.4	-1.9	-	-0.5	-	2.7	2.4	0.1	0.5	0.1	-	0.3	1.5	2.5	-
**NorthPeruCoast_1750-560BP (EIP-LIP)**	-	-	3.8	3.9	-2.4	-	-0.8	2.1	2.9	3.4	-1.1	-	-1.3	-	3.1	-1.1	0.5	1.0	-3.0	-	-1.0	0.3	2.8	-
**NorthPeruHighlands_1200-550BP (MH-LIP)**	-	0.1	-	2.5	-2.7	-	-0.7	1.1	1.7	2.9	-2.3	-	-2.5	-	0.2	-1.1	1.1	2.6	-1.6	-	-0.3	0.5	2.4	-
**NorthPeruHighlands_LaGalgada_4100BP (LA)**	-	-0.4	1.0	-	-3.3	-	-0.6	-0.5	1.9	2.7	-1.3	-	-1.1	-	1.5	-1.6	1.0	1.6	-1.3	-	-1.4	1.0	3.2	-
**NorthPeruHighlands_Lauricocha_8600BP (EA)**	-	-6.6	-4.4	-3.4	-	-	0.5	-5.4	-1.4	0.1	-0.6	-	-2.0	-	0.5	-0.3	0.3	1.7	-1.1	-	-0.8	-0.2	2.4	-
**NorthPeruHighlands_Lauricocha_5800BP (MA)**	-	-	-	-	-	-	-	-	0.0	1.9	-0.1	-	-1.6	-	-0.2	0.8	0.4	1.1	-1.5	-	-1.0	-2.1	0.9	-
**NorthPeruHighlands_Lauricocha_3500BP (IP)**	-	-7.8	-5.7	-3.8	-3.0	-	-	-6.6	-0.9	0.4	-1.3	-	-2.8	-	1.1	1.1	2.0	1.9	0.2	-	0.3	-0.8	2.9	-
**CentralPeruCoast_1850-480BP (EIP-LIP)**	-	-0.6	2.2	1.7	-3.2	-	-1.1	-	3.0	3.8	-0.6	-	-0.2	-	2.2	-1.2	1.5	2.7	-2.6	-	-1.6	-0.2	3.7	-
**SouthPeruCoast_1480-515BP (EIP-LIP)**	-	2.0	4.0	3.0	-1.7	-	0.2	1.8	-	4.9	-2.1	-	-1.1	-	-2.0	-3.1	2.4	4.9	-1.1	-	0.6	2.5	3.0	-
**SouthPeruHighlands_1150-390BP (MH-LH)**	-	0.2	3.5	2.5	-2.0	-	-0.5	1.4	2.5	-	-2.7	-	-1.0	-	-1.9	-2.1	2.1	6.5	-2.2	-	-1.7	-0.4	3.2	-
**Cuncaicha_9000BP (EA)**	-	-1.3	0.1	-0.2	-0.6	-	-0.5	-0.6	-1.6	-0.4	-	-	-1.5	-	-0.2	0.7	1.7	0.0	-0.6	-	0.7	0.2	2.8	-
**Cuncaicha_4200BP (LA)**	-	-0.1	1.9	0.8	0.1	-	0.4	-0.3	-	-	-	-	-	-	-2.7	1.1	-0.9	-2.7	-0.1	-	-1.5	-2.5	0.9	-
**Cuncaicha_3300BP (IP)**	-	0.8	1.6	1.3	-0.1	-	-0.6	1.4	-4.2	-2.9	-4.4	-	-	-	-4.1	-1.1	-1.2	1.1	-0.2	-	-0.9	-0.9	1.9	-
**Cusco_SanSebastian_610-490BP (LIP-LH)**	-	-1.0	2.1	0.9	-1.8	-	-0.8	0.7	-0.8	-0.2	-3.4	-	-2.1	-	-	-	1.4	1.8	-1.6	-	-2.8	-1.1	1.1	-
**Cusco_Torontoy_A_450BP (LH)**	-	1.3	2.4	1.9	-2.0	-	0.8	2.6	0.2	1.5	-1.9	-	-3.9	-	-	-0.4	-1.1	1.4	-2.8	-	-2.6	-1.3	3.4	-
**Cusco_Torontoy_B_450BP (LH)**	-	-1.9	0.0	-1.2	-2.3	-	0.5	-1.2	-4.0	-2.3	-3.3	-	-4.0	-	-0.7	-	-2.5	-4.6	-2.8	-	6.0	-3.4	2.4	-
**TiticacaBasin_Tiwanaku_1000BP (MH)**	-	-0.2	0.9	1.2	-1.6	-	-0.7	0.6	-2.5	-1.0	-3.1	-	-3.2	-	-4.0	0.0	-	-1.1	-1.6	-	-2.1	-4.2	1.7	-
**TiticacaBasin_Chile_Pukara_700BP (LIP)**	-	0.1	2.1	1.5	-0.9	-	0.7	1.0	1.9	4.9	-1.9	-	0.2	-	-2.4	-3.1	-0.4	-	-1.5	-	-0.9	-0.2	2.0	-
**TiticacaBasin_RioUncallane_1700BP (EIP)**	-	-0.8	1.1	1.1	-1.7	-	-0.3	0.2	-7.5	-5.8	-6.5	-	-4.6	-	-3.5	2.6	-2.8	-4.6	-	-	-3.4	-6.5	4.3	-
**TiticacaBasin_MiraFlores_1100BP (MH)**	-	0.0	1.1	1.2	-2.2	-	-1.3	0.7	-6.2	-4.0	-5.3	-	-3.8	-	-3.3	3.8	-	-	-	-	-	-	3.4	-
**TiticacaBasin_Iroco_1050BP (MH)**	-	0.5	1.8	0.4	-1.2	-	-0.4	0.3	-2.5	-2.5	-2.3	-	-2.6	-	-1.2	10	-1.0	-1.7	-1.4	-	-	-0.7	2.4	-
**NorthChile_1350-570BP (MH-LIP)**	-	1.5	2.2	3.5	-0.5	-	-0.7	1.8	-1.9	-1.6	-3.5	-	-2.7	-	-2.4	1.1	-0.6	1.5	1.0	-	1.9	-	2.8	-
**Argentina_LagunaChica_1600BP**	-	0.6	0.8	1.2	-1.1	-	-0.8	1.5	0.0	1.0	-0.1	-	-0.5	-	1.9	0.9	-1.0	-0.1	-1.7	-	-1.0	0.1	-	-
**Argentina_LagunaChica_8600BP**	-	0.4	0.7	-0.1	1.2	-	-2.0	0.2	-1.4	0.4	-1.9	-	-1.9	-	-1.0	-0.8	0.5	0.6	-2.9	-	-0.8	1.0	-	-
