# Supplementary material for: A Paleogenomic Reconstruction of the Deep Population History of the Andes
Source: Cell. 2020 May 28;181(5):1131–1145.e21. doi: 10.1016/j.cell.2020.04.015 (PMC7304944; doi:10.1016/j.cell.2020.04.015)
Supplement: Data S1 — English and Spanish Translation of the Summary and Key Findings Sections, Related to STAR Methods [file mmc9.pdf]

Data S1.

English and Spanish translation of the Summary and Key Findings sections. Related to Figure STAR Methods.

Box 1: Summary of Main Findings

The Nature of Population Structure

1. Population Structure Has Early Holocene Roots

The population structure that distinguishes Highland groups from other South Americans was in place by ~9000 BP. A North vs. South population structure developed by at least ~5800 BP and correlates strongly with North vs. South structure today.

2. Gene Flow after the Establishment of Population Structure

Prior to ~2000 BP, gene flow occurred between the North Peru Highlands and Coast, and between the North Peru Highlands and the South Peru Highlands and Coast.

3. Continuity in Most Regions after ~2000 BP

The population structure that developed by ~2000 BP largely persisted through major cultural and sociopolitical changes with Indigenous peoples today and just prior to European contact tending to be most closely related to ~2000BP groups from the same regions. However, we also detect two important exceptions to this continuity.

4. Cosmopolitanism During the Tiwanaku and Inca Periods

During the development of the Tiwanaku culture, the sampled individuals from Tiwanaku, the polity's administrative center, were more closely related to South Peru Highlands individuals than were people in other areas of Lake Titicaca Basin. During the Inca expansion we detect ancestry heterogeneity in the Cusco region and long-distance movement of individuals in their lifetimes.

Interactions Beyond the Andes

5. Gene Flow Between the Northwest Amazon and North Peru

Gene flow occurred between the Northwest Amazon and North Peru, with more affinity of Amazonians with groups on the North Peru Coast than the Highlands.

6. Gene Flow Between the Argentine Pampas and South-Central Andes

There was genetic exchange between North Chile and South Peru with at least some people living in the Argentine Pampas in the period ~6800-1600 BP.

7. Distinctive Ancestry Profile that Arrived by ~4200 BP Integrated by ~2000 BP

Previous work showed that by ~4200 BP, some Central Andeans harbored ancestry more closely related to ancient people of the California Channel Islands than is the case for other South Americans, reflecting complexity in the peopling of South America. This ancestry was fully integrated across the Central Andes by ~2000 BP.

Una Reconstrucción Paleogenómica de la Historia Profunda de la Población Andina

En Breve
Se reportan los cambios genéticos de las poblaciones en los Andes centrales a lo largo de 9000 años en base a datos de genomas completos correspondientes a 89 individuos antiguos, revelando flujo génico a gran escala y la naturaleza cosmopolita de las sociedades Tiwanaku e Inca.

Destacados

- Un gradiente de ADN antiguo revela el desarrollo de una subestructura de norte-sur en las tierras altas (o sierras) de los Andes a partir de 5800 años atrás.
- Después de 5800 años AP, se observa flujo génico entre las poblaciones de la sierra y sus vecinos.
- Después de 2000 años AP, se observa notable continuidad genética a través del desarrollo y desintegración de mayores formaciones culturales.

Resumen
Existen numerosas preguntas sin respuesta acerca de la historia de la población de los Andes centrales y sur-centrales, particularmente con respecto al impacto de las formaciones sociales de gran escala, como lo fueron los Mochica, Wari, Tiwanaku e Inca. En este estudio, recopilamos datos de genomas completos de 89 individuos que datan entre ~ 9000 y 500 años antes del presente (AP), con un enfoque particular en el período del auge y la caída de las sociedades estatales. La estructura genética que existe hoy en día comenzó a desarrollarse hace 5800 AP, seguida por el flujo génico bidireccional entre la Sierra del Norte y del Sur, y entre la Sierra y la Costa. Detectamos una mezcla mínima entre grupos vecinos entre ~2000 y 500 AP, aunque detectamos cosmopolitismo (personas de diversos ancestros que viven lado a lado) en el núcleo de las sociedades Tiwanaku e Inca. También revelamos casos de movilidad de largo alcance que conectan los Andes con las pampas argentinas, y los Andes del noroeste con la cuenca del Amazonas.

Cuadro 1. Resumen de hallazgos principales

La Naturaleza de la Estructura Poblacional en los Andes

La estructura poblacional tiene raíces en el Holoceno temprano

1. La estructura poblacional en los Andes tiene raíces durante el Holoceno Temprano.
   La estructura poblacional que distingue a los grupos andinos de tierras altas de otros sudamericanos se estableció hace aproximadamente ~9000 AP. Una estructura poblacional Norte vs. Sur se desarrolló al menos hace ~5800 años atrás y se encuentra fuertemente correlaciona con la estructura Norte vs. Sur existente hoy en día.
2. Flujo genético después del establecimiento de la estructura poblacional.
   Antes de ~2000 AP, flujo genético ocurrió entre las poblaciones de la sierra y la costa del norte de Perú, y entre las poblaciones de la sierra norte y sur del Perú.
3. Continuidad en la mayoría de las regiones después de ~2000 AP.
   La estructura poblacional que se desarrolló a partir de ~2000 AP persistió en gran medida a través de importantes cambios culturales y sociopolíticos y como resultado, los pueblos

indígenas de hoy como aquellos presentes justo antes de la conquista europea, tienden a estar más estrechamente relacionados con los grupos de hace ~2000 AP en sus mismas regiones. Sin embargo, también detectamos dos excepciones importantes a esta continuidad.

4. Cosmopolitismo durante los períodos Tiwanaku e Inca.
   Durante el desarrollo de la cultura Tiwanaku, los individuos muestreados del sitio de Tiwanaku, el centro administrativo estatal, estuvieron más estrechamente relacionados con los individuos de la sierra sur del Perú que con individuos de otras áreas de la cuenca del Lago Titicaca. Durante la expansión Inca detectamos heterogeneidad ancestral en la región de Cusco y movimientos de personas a larga distancia durante sus vidas.

Interacciones más allá de los Andes

5. Flujo genético entre el noroeste de la Amazonía y el norte del Perú
   El flujo génico entre el noroeste de la Amazonía y el norte del Perú tuvo más afinidad entre los amazónicos con los grupos de la costa norte del Perú que con aquellas de la sierra.
6. Flujo génico entre la pampa argentina y los Andes centro-sur
   Hubo un intercambio genético entre poblaciones del norte de Chile y el sur del Perú con al menos algunos pobladores de la pampa argentina en el período de ~6800 a 1600 AP.
7. Un perfil de ascendencia distintivo se desarrolló hace ~4200 AP y se integró hace ~2000 AP
   Investigaciones previas demostraron que hace ~4200 AP, algunos individuos de los Andes centrales albergaban ascendencia más estrechamente relacionada con los antiguos pobladores de las Islas del Canal en California que el resto de los sudamericanos, lo que refleja la complejidad de la población de América del Sur. Esta ascendencia se integró por completo en los Andes centrales hace ~2000 AP.
